# Rhomboids of Mycobacteria: Characterization Using an *aarA* Mutant of *Providencia stuartii* and Gene Deletion in *Mycobacterium smegmatis*

**DOI:** 10.1371/journal.pone.0045741

**Published:** 2012-09-21

**Authors:** David Patrick Kateete, Fred Ashaba Katabazi, Alfred Okeng, Moses Okee, Conrad Musinguzi, Benon Byamugisha Asiimwe, Samuel Kyobe, Jeniffer Asiimwe, W. Henry Boom, Moses Lutaakome Joloba

**Affiliations:** 1 Department of Medical Microbiology, School of Biomedical Sciences, Makerere University College of Health Sciences (MakCHS), Kampala, Uganda; 2 College of Veterinary Medicine, Animal Resources and Biosecurity (CoVAB), Makerere University, Kampala, Uganda; 3 Tuberculosis Research Unit (TBRU), Case Western Reserve University, Cleveland, Ohio, United States of America; University of Padova, Italy

## Abstract

**Background:**

Rhomboids are ubiquitous proteins with unknown roles in mycobacteria. However, bioinformatics suggested putative roles in DNA replication pathways and metabolite transport. Here, mycobacterial rhomboid-encoding genes were characterized; first, using the *Providencia stuartii* null-rhomboid mutant and then deleted from *Mycobacterium smegmatis* for additional insight in mycobacteria.

**Methodology/Principal Findings:**

Using in silico analysis we identified in *M. tuberculosis* genome the genes encoding two putative rhomboid proteins; Rv0110 (referred to as “rhomboid protease 1”) and Rv1337 (“rhomboid protease 2”). Genes encoding orthologs of these proteins are widely represented in all mycobacterial species. When transformed into *P. stuartii* null-rhomboid mutant (Δ*aarA*), genes encoding mycobacterial orthologs of “rhomboid protease 2” fully restored AarA activity (AarA is the rhomboid protein of *P. stuartii*). However, most genes encoding mycobacterial “rhomboid protease 1” orthologs did not. Furthermore, upon gene deletion in *M. smegmatis*, the ΔMSMEG_4904 single mutant (which lost the gene encoding MSMEG_4904, orthologous to Rv1337, “rhomboid protease 2”) formed the least biofilms and was also more susceptible to ciprofloxacin and novobiocin, antimicrobials that inhibit DNA gyrase. However, the ΔMSMEG_5036 single mutant (which lost the gene encoding MSMEG_5036, orthologous to Rv0110, “rhomboid protease 1”) was not as susceptible. Surprisingly, the double rhomboid mutant ΔMSMEG_4904–ΔMSMEG_5036 (which lost genes encoding both homologs) was also not as susceptible suggesting compensatory effects following deletion of both rhomboid-encoding genes. Indeed, transforming the double mutant with a plasmid encoding MSMEG_5036 produced phenotypes of the ΔMSMEG_4904 single mutant (i.e. susceptibility to ciprofloxacin and novobiocin).

**Conclusions/Significance:**

Mycobacterial rhomboid-encoding genes exhibit differences in complementing *aarA* whereby it's only genes encoding “rhomboid protease 2” orthologs that fully restore AarA activity. Additionally, gene deletion data suggests inhibition of DNA gyrase by MSMEG_4904; however, the ameliorated effect in the double mutant suggests occurrence of compensatory mechanisms following deletion of genes encoding both rhomboids.

## Introduction

The emergence of unusual drug resistant strains of *Mycobacterium tuberculosis* (*MTB*) threatens to make tuberculosis (TB) incurable again [1], [2]. This underscores the need for new or improved anti-TB drugs and vaccines, or diagnostics. However, achieving this usually requires elucidating the complex biology of the causative agent, *Mycobacterium tuberculosis* complex (MTBC) [3], [4].

Rhomboids are novel proteins that occur widely in bacteria and are currently the subject of intense investigation [5], [6], [7], [8], [9], [10], [11]. However, little is known about them in the MTBC species and in mycobacteria at large. Besides, many genomes for unicellular and multicellular organisms contain rhomboid-like genes, widely annotated to encode unusual membrane-bound serine proteases conserved across kingdoms [5], [6], [7]. While they are so far known to occur only in cellular forms, the giant viral genomes of *Acanthamoeba* also possess genes encoding proteins with rhomboid domains [http://www.uniprot.org/uniprot/Q5UQ86].

Rhomboid proteins are believed to participate in universal signaling mechanisms yet to be elucidated [5], [6]. Originally discovered in Drosophila (being named after an altered rhombus-like appearance of a mutant embryo following disruption of an unknown gene which investigators termed ‘*rho*’ [11]), AarA of the urinary tract pathogen *Providencia stuartii* is presumably the most characterized rhomboid. AarA is involved in an unusual type of quorum sensing in this Gram-negative bacterium. Additionally, AarA activity in the null-rhomboid mutant of *P. stuartii* (Δ*aarA*) can be restored by rhomboid-encoding genes of evolutionary diverse species [5], [10], [12].

While rhomboids are widely conserved, they also display specific roles in diverse species: regulation of mitochondrial function in humans [5], [7]; signaling by the epidermal growth factor receptor (EGFR) which controls homeostasis, wing venation and proper eye development in *Drosophila melanogaster* [5], [12]; and red blood cell invasion by *Plasmodium falciparum* [5], [13]. These have attracted attention as useful targets for development of new drugs, vaccines and other medical interventions [5], [7], [9]. Despite this, functions are ascribed only to a few and roles of rhomboids are largely unknown in most species [5].

Most mycobacterial species possess two rhomboid-encoding genes that have not been experimentally characterized [14]. Recently however, bioinformatics analyses suggested that mycobacterial rhomboids could have roles in DNA replication pathways or metabolite transport [14]. Here we aimed to characterize rhomboid-encoding genes in mycobacteria and scrutinize their phenotypes in established systems for insight into roles in physiology. A two systematic approach was followed:

- First, rhomboid-encoding genes were cloned from diverse species of mycobacteria, pathogenic and non-pathogenic. Then, each recombinant rhomboid-encoding gene from mycobacteria was characterized for phenotype rescue ability in the *P. stuartii* system popular for studying rhomboid activity [15].
- Second, to further decipher their role(s) in mycobacteria, we genetically deleted the two rhomboid-encoding genes (*MSMEG_4904* and *MSMEG_5036*) in *Mycobacterium smegmatis* and phenotypes of the single and double rhomboid mutants characterized.

## Results and Discussion

Using in silico analysis we previously identified in *M. tuberculosis* genome the genes encoding two putative rhomboid proteins; Rv0110 and Rv1337. Genes encoding orthologs of these proteins are widely represented in all mycobacteria. In this text, we have for simplicity categorized proteins encoded by these genes as “rhomboid protease 1” (representing mycobacterial rhomboids orthologous to Rv0110) and “rhomboid protease 2” (representing mycobacterial rhomboids orthologous to Rv1337) [14] (see **Table 1** for details).

**Table 1:** Distribution of “rhomboid protease 1” and “rhomboid protease 2” in selected mycobacterial species/strains.

	Rhomboid protease 1^a^			Rhomboid protease 2^b^		
Species	Protein ID [Accession #]	Length	Annotation	Protein ID [Accession #]	Length	Annotation
*M. tuberculosis* (H_37_Rv)	Rv0110 [NP_214624.1]	249	Hypothetical protein	Rv1337 [NP_215853]	240	Hypothetical protein
*M. bovis* (BCG)	BCG_0143 [YP_976246.1]	249	Integral membrane protein	BCG_1399 [YP_977491.1]	240	Putative integral membrane protein
*M. bovis*	Mb0114 [NP_853781.1]	249	Hypothetical protein	Mb1372 [NP_855026.1]	240	Hypothetical protein
*M. africanum*	MAF_01110 [YP_004721836.1]	249	Hypothetical protein	MAF_13610 [YP_004723048.1]	240	Hypothetical protein
*M. canettii*	MCAN_01131 [YP_004743597.1]	249	Putative integral membrane protein	MCAN_13551 [YP_004744805.1]	240	Putative integral membrane protein
*M. marinum*	MMAR_0300 [YP_001848622.1]	289	Serine protease	MMAR_4059 [YP_001852321.1]	222	Integral membrane protein
*M. ulcerans*	MUL_4822 [YP_908200.1]	254	Hypothetical protein	MUL_3926 [4553595]	-	Pseudogene
*M. leprae*	-	-	-	ML1171 [NP_301853.1]	238	Hypothetical protein
*M. avium*	-	-	-	MAV_1554 [YP_880789.1]	223	Rhomboid family protein
MAP^c^	-	-	-	MAP2425c [NP_961359.1]	147	Hypothetical protein
MAP	-	-	-	MAP2426c [NP_961360.1]	72	Hypothetical protein
*M. intracellulare*	-	-	-	OCQ_13860 [YP_005342302.1]	223	Rhomboid family protein product
*M. colombiense*	-	-	-	[ZP_08714662.1]	203	Rhomboid family protein
*M. xenopi*	-	-	-	MXEN_10301 [ZP_09980385.1]	219	Integral membrane protein
*M. kansasii*	[ZP_04746743.1]	288	Integral membrane protein	[ZP_04748139.1]	222	Integral membrane protein
*M. rhodesiae*	MycrhN_1673 [YP_004999502.1]	248	Peptidase S54, rhomboid domain protein	MycrhN_3867 [YP_005001593.1]	220	Peptidase S54, rhomboid domain protein
*M. smegmatis*	MSMEG_5036 [YP_889287.1]	250	Rhomboid family protein	MSMEG_4904 [YP_889159.1]	219	Rhomboid family protein
*M. phlei*	MPHLEI_19664 [ZP_09976832.1]	248	hypothetical protein	[ZP_09976766.1]	209	Rhomboid family protein
*M. tusciae*	[ZP_09681852.1]	282	Rhomboid family protein	[ZP_09684123.1]	231	Rhomboid family protein
*M. gilvum*	Mflv_1071 [YP_001132341]	279	Hypothetical protein	Mflv_2355 [YP_001133621.1]	225	Rhomboid family protein
*M. thermoresistibile*	[ZP_09081357.1]	281	Rhomboid family protein	KEK_13008 [ZP_09083165.1]	226	Hypothetical protein
*M. vanbaalenii*	Mvan_5753 [YP_956524.1]	290	Rhomboid family protein	Mvan_4290 [YP_955073.1]	225	Rhomboid family protein

a Genes encoding “rhomboid protease 1” are missing in *M. leprae* and the *M. avium* complex (MAC) [14].

b the gene encoding “rhomboid protease 2” in *M. ulcerans* is a pseudogene (Gene ID number indicated);

c *M. avium subsp. paratuberculosis*. Annotation and gene/protein names are as described in KEGG (Kyoto Encyclopedia of Genes and Genomes [http://www.genome.jp/kegg/]) or from the respective genomes. See **Table S1** for details.

Inactivation of *aarA*, the rhomboid-encoding gene of *P. stuartii*, yields the following pleiotropic phenotypes; a) absence of the golden yellow pigment in colonies, b) inability of the *P. stuartii* null-rhomboid mutant to grow on MacConkey agar, c) absence of an assayable putative extracellular signal that regulates cellular functions, and d) defects in cell division (that manifest as abnormality in cell morphology in which the mutant assumes filamentous, paired, sickle or coccobacillary appearance) [10], [15]. Although the precise mechanisms for these defects have not been described, by assaying for each of them, the *P. stuartii* null-rhomboid mutant (i.e. an *aarA* deficient strain, XD37.A) has been useful in assessing the activity of evolutionary diverse rhomboid-encoding genes [6], [15]. Using this model, we determined whether the genes encoding mycobacterial rhomboids would also restore AarA activity.

### Mycobacterial rhomboid-encoding genes exhibit differences in complementing *aarA*: it's only genes encoding “rhomboid protease 2” orthologs that fully complement AarA activity

**Figure 1:**
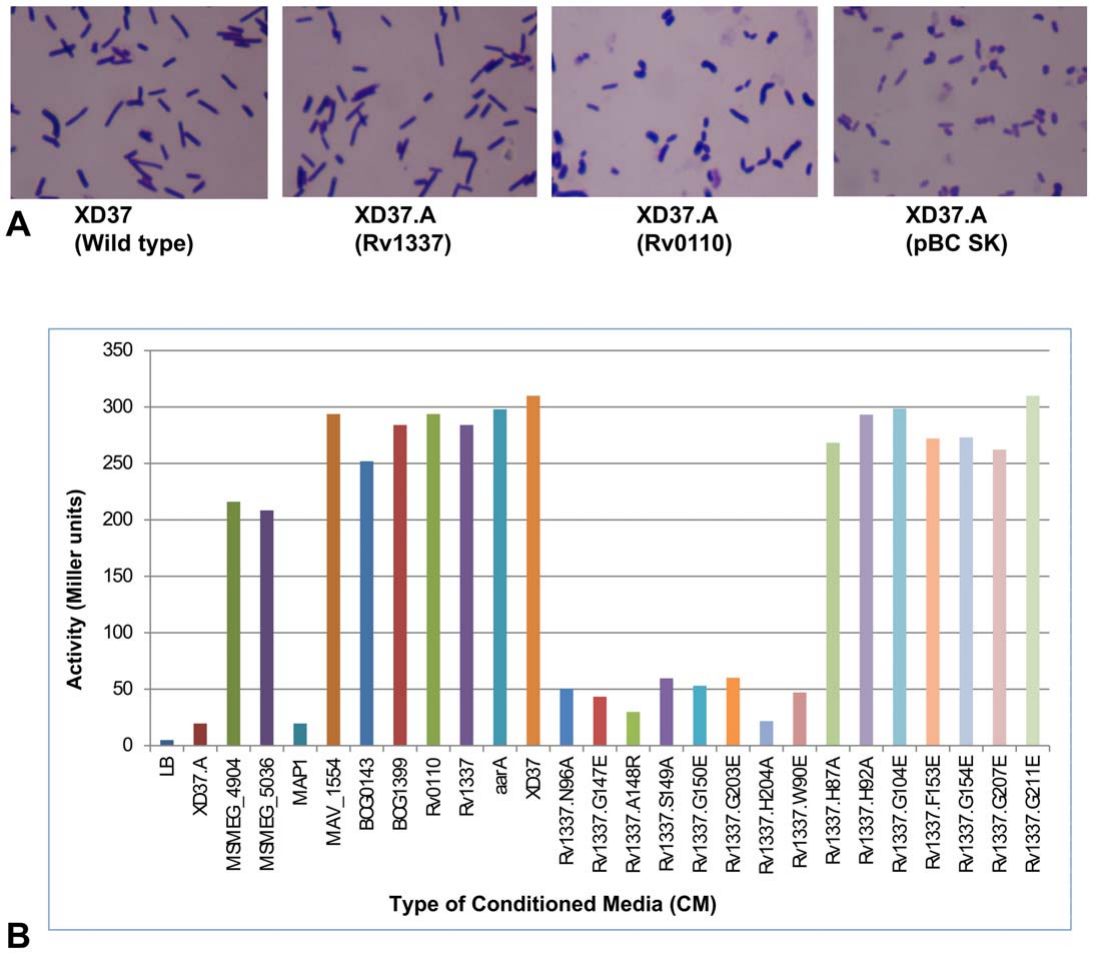
Mycobacterial rhomboid-encoding genes exhibit differences in complementing *aarA*. **Panel A:** Genes encoding mycobacterial orthologs of “rhomboid protease 2” (orthologous to Rv1337) fully restored the wild type cell morphology (rods) in XD37.A. On the other hand, the phenotype of XD37.A transformed with plasmids encoding orthologs of “rhomboid protease 1” (orthologous to Rv0110) from MTBC was similar to that of the uncomplemented mutant (XD37.A transformed with the parent plasmid pBCSK). **Panel B:** β-galactosidase assays showing restoration of the putative extracellular signal in XD37.A transformed with several plasmids encoding mycobacterial rhomboids. The genes encoding the hypothetical proteins of *Mycobacterium avium* subspp. *paratuberculosis* (MAP1), which lack the resdiue (W90), did not restore the putative signal. Genes encoding rhomboids of the MTBC and *M. avium* were the strongest at complementing the putative signal. Panel B also shows that mutation in codons of the rhomboid family residues (W90, N96, G147, A148, S149, G150, G203 and H204) of Rv1337 abolishes production of the putative signal (as well as complementing other phenotypes ascribed to *aarA*). Mutations of codons of other residues with no effect on complememntation of signal production are also shown (H87A, H92A, G104Q, F153S, G154Q and G207Q), as well as G211E, which instead intensifed complementation. Apart from the codon for G211, all the inactivated codons resulted in failed complementation in terms of pigment production and cell morphology (not shown). Each data point was from an average of four experiments.

With exception of the rhomboid encoding genes of MAP (*M. avium* subspecies *paratuberculosis*, MAP2425c and MAP2426c), all the genes encoding mycobacterial orthologs of “rhomboid protease 2” fully complemented the pleiotropic phenotypes ascribed to loss of *aarA* (**Figure 1** **, panel a, and** **Table 2**). In this regard, these genes can be assumed to be functionally equivalent to *aarA*.

**Table 2:** Mycobacterial rhomboid-encoding genes display differences in complementing *aarA*.

		Phenotype rescue ability			
Rhomboid	Accession# (GenBank)	Pigment production	Cell morphology	Growth on MacConky	Signal production
XD37^a^	**-**	Yes	rods	Yes	Yes
XD37.A^b^	**-**	No	chains	No	No
XD37.A+*aarA* c	L28755	Yes	rods	Yes	Yes
***Rhomboid protease 1***					
XD37.A+Rv0110 [H37Rv]	HM453890	No	chains	No	Yes
XD37.A+Rv0110 [BN44]	HM453892	No	chains	No	Yes
XD37.A+BCG_0143 [BCG]	HM453894	No	chains	No	Yes
XD37.A+Mb0114 [JN55]	HM453896	No	chains	No	Yes
XD37.A+MSMEG_5036	HM453900	Yes	rods	Yes	Yes
***Rhomboid protease 2***					
XD37.A+Rv1337 [H37Rv]	HM453891	Yes	rods	Yes	Yes
XD37.A+Rv1337 [BN44]	HM453897	Yes	rods	Yes	Yes
XD37.A+BCG1399 [BCG]	HM453895	Yes	rods	Yes	Yes
XD37.A+Mb1374 [JN55]	HM453897	Yes	rods	Yes	Yes
XD37.A+MAV_1554	HM453898	Yes	rods	Yes	Yes
XD37.A+MAP2425c	NP_961359	No	chains	No	No
XD37.A+MAP2426c	NP_961360	No	chains	No	No
XD37.A+MAP2425c/2426c^e^	HM453899	No	chains	No	No
XD37.A+MSMEG_4904	HM453901	Yes	rods	Yes	Yes
***Effect of mutating residues typical of rhomboids on complementation***					
XD37.A+Rv1337.L86N	-	No	chains	Yes	Yes
XD37.A+Rv1337.L87N	-	No	chains	Yes	Yes
XD37.A+Rv1337.H87A	-	No	chains	Yes	Yes
XD37.A+Rv1337.H92A	-	No	chains	Yes	Yes
XD37.A+Rv1337.W90E	-	No	chains	No	No
XD37.A+MAV1554.W90E	-	No	chains	No	No
XD37.A+Rv1337.N96A	-	No	chains	No	No
XD37.A+Rv1337.G104E	-	No	chains	Yes	Yes
XD37.A+Rv1337.G147E	-	No	chains	No	No
XD37.A+Rv1337.A148R	-	No	chains	No	No
XD37.A+Rv1337.S149A	-	No	chains	No	No
XD37.A+Rv1337.G150E	-	No	chains	No	No
XD37.A+Rv1337.F153S	-	No	chains	Yes	Yes
XD37.A+Rv1337.G154E	-	No	chains	Yes	Yes
XD37.A+Rv1337.G203E	-	No	chains	No	No
XD37.A+Rv1337.H204A	-	No	chains	No	No
XD37.A+Rv1337.G207E	-	No	chains	Yes	Yes
XD37.A+Rv1337.G211E	-	Yes	rods	Yes	Yes

This table summarizes phenotype rescue ability in XD37.A by mycobacterial rhomboid-encoding genes. With the exception of *MAP2426c* and *MAP2425c*, all genes encoding the orthologs of “rhomboid protease 2” fully restored AarA activity (i.e. the golden yellow pigment of colonies, cell morphology, growth on MacConkey agar and the putative signal). *MSMEG_5036* was the only “rhomboid protease 1” encoding gene that behaved like genes encoded by “rhomboid protease 2” but comparatively weaker. However, all mycobacterial rhomboid-encoding genes (except those of those of MAP) strongly activated production of the extracellular putative signal. Species/strains from which rhomboids were cloned are indicated in brackets (*MTB* H_37_Rv, *MTB* clinical isolates [BN44], *M. bovis* BCG, *M. bovis* cattle strain [JN55], *M. avium* clinical isolate [SU-36800], MAP and *M. smegmatis* [SMR5]).

a *P. stuartii* wild type strain;

b *P. stuartii* rhomboid deficient strain;

c *P. stuartii* rhomboid deficient strain complemented with *aarA* (wild type gene);

e Two hypothetical rhomboid gene fragments (encoding MAP2426c and MAP2425c) cloned in-frame;

**-**, not applicable.

On the other hand, the genes encoding mycobacterial orthologs of “rhomboid protease 1” were weak in complementing *aarA*. For the MTBC species, these genes only complemented production of a putative extracellular signal; the golden yellow pigment of colonies, cell morphology and growth on MacConkey agar were not restored. Indeed, *MSMEG_5036* of *M. smegmatis* was the only gene encoding a mycobacterial ortholog of “rhomboid protease 1” that fully complemented *aarA* albeit weakly (**Table 2**). Therefore, the only phenotype convincingly rescued by the genes encoding mycobacterial “rhomboid protease 1” orthologs was production of the putative signal (**Figure 1**, **panel a, and** **Table 2**).

While there's no apparent explanation for the discrepancy above in complementing *aarA* by mycobacterial rhomboid-encoding genes, evolutionary divergence may contribute. For instance, the orthologs of “rhomboid protease 2” are well conserved in mycobacteria yet those of “rhomboid protease 1” are not and are missing in the MAC species and *M. leprae* [14]. However, rhomboid-encoding genes stimulating production of a putative signal but failing to restore the golden yellow pigment in colonies, cell morphology and growth on MacConkey agar, have been previously reported [15].

### Rv1337 with inactivated residues typical of rhomboid proteins does not complement AarA activity

Most rhomboid proteins possess the following residues at their C-termini (numbering based on Rv1337, accession # NP_215853): H87, H92, N96, G104, G147, A148, G150, S149, G154, G203, H204 and G207 [14], [16]. Of these, S149 and H204 form the “catalytic dyad” responsible for proteolysis among “active rhomboids” (active rhomboids possess residues S149 and H204 in their transmembrane helices [8], [14]). However, catalysis has also been demonstrated for the “inactive rhomboids” [17] (these lack residues S149 and H204) [8]. Thus, predicting functionality of rhomboids based on the presence or absence of residues typical of rhomboids may be insufficient [17].

Using the Rv1337-encoding gene as a prototype, we mutated through site directed mutagenesis (SDM) codons for rhomboid domain residues to map those contributing to complementation of *aarA*. *Rv1337* gene-mutants (in plasmid pBCSKrho2D) with mutated codons were individually transformed into *P. stuartii* null-rhomboid mutant to determine the effect of mutation on complementation of *aarA*.

#### i) Residues responsible for restoring pigment production and cell morphology

When codons for the following residues; H87, H92, G104, G154 and G207 in Rv1337 were mutated, the golden yellow pigment of colonies and cell morphology were no longer restored by the Rv1337-encoding gene when transformed into rhomboid deficient *P. stuartii*. However, growth on MacConkey agar and production of a putative signal still occurred (**Figure 1** **, panel b**). Thus, these residues partially contribute to complementation of *aarA*.

#### ii) Residues responsible for full complementation of *aarA*

Here full complementation refers to the presence of all the pleiotropic phenotypes ascribed to *aarA* (i.e. the golden yellow pigment of colonies, cell morphology, growth on MacConkey agar and production of a putative signal) following transformation of rhomboid-deficient *P. stuartii* with a rhomboid encoding plasmid.

When codons for the following residues; N96, G147, A148, S149, G150, G203 and H204 were mutated, the Rv1337-encoding gene could no longer complement any of the *aarA* pleiotropic phenotypes in rhomboid deficient *P. stuartii*, -**Figure 1** **, panel b**. Hence, these residues are essential for full complementation of *aarA*.

Similar results were achieved in previous studies where the codons for S149 and H204 (rhomboid catalytic dyad) were inactivated in rhomboid-encoding genes of *Escherichia coli* and *Pseudomonas aeruginosa* [15], [16].

### W90 (a non-rhomboid residue) is essential for complementation of AarA activity

Most orthologs of mycobacterial “rhomboid protease 1” and “rhomboid protease 2” analyzed so far are “active proteases” in that they possess the catalytic dyad [8] [14]. However, from the data described above, it was surprising that genes encoding them exhibit differences in complementing *aarA*.

We postulated occurrence in mycobacterial rhomboids of additional residues or motifs contributing to differences in complementing AarA activity. Three residues; W90, F153 and G211, were identified through sequence analysis. F153 (an active site stabilizing residue) and G211 do occur widely in both rhomboid types of mycobacteria. However, W90 is missing in “rhomboid protease 1” and appears unique to “rhomboid protease 2” [14].

Using the Rv1337-gene encoding as a template, we mutated through SDM the codons for W90, F153 and G211 to determine whether they contribute to complementation of *aarA*. The mutation F153S only abrogated restoration of the golden yellow pigment of colonies and cell morphology typical of wild type *P. stuartii*; growth on MacConkey agar and signal production still occurred. On the other hand, G211E intensified AarA activity (i.e. in comparison with the wild type Rv1337-encoding gene, the gene mutant encoding Rv1337.G211E was better at complementing *aarA*).

On the other hand, the mutation W90Q fully abrogated complementation of AarA activity in similar proportion to the seven residues discussed above (i.e. the golden yellow pigment, cell morphology, growth on MacConkey agar and a putative signal were no longer restored in rhomboid deficient *P. stuartii* transformed with a plasmid encoding Rv1337.W90Q, **figure 1** **, panel b**). Notably, W90 is not a rhomboid domain residue and there's so far no role ascribed to it. Similar results were obtained for MAV_1554 of *M. avium* following mutation of the W90 codon. Thus, W90 could be physiologically important in most mycobacterial orthologs of rhomboid protease 2.

Overall, with the exception of G211, mutation of codons for seventeen residues in the Rv1337-encoding gene was found essential in restoring pigment production and cell morphology in XD37.A. Of these however, only eight were concomitantly essential for restoring growth on MacConkey agar and a putative signal (**Table 2**). Therefore, it is these eight residues (W90, N96, G147, A148, S149, G150, G203 and H204) that are fully essential in complementing AarA activity (**Table 2**).

### Absence of W90 could be responsible for failed complementation by the MAP rhomboid encoding genes

MAP has two small hypothetical proteins with similarity to rhomboids: MAP2426c and MAP2425c. MAP2425c (147 residues) is identical to the C-terminus of MAV1554 (the rhomboid protein of *M. avium*, 223 residues). MAP2425c possesses rhomboid family residues including the catalytic dyad. On the other hand, MAP2426c is shorter (72 residues) and identical to the N-terminal of MAV_1554. MAP2426c lacks rhomboid family residues. MAP and *M. avium* belong to a group of genetically related organisms referred to as the *Mycobacterium avium* complex (MAC) which share approx. 98% DNA sequence identity [18].

MAP2426c, MAP2425c and MAV1554 are orthologs of “rhomboid protease 2” [14]. Interestingly, the gene encoding MAV_1554 unequivocally complemented *aarA* in similar proportion to the Rv1337-encoding gene. It was thus puzzling that none of the MAP rhomboid-encoding genes (*MAP2426c* and *MAP2425c*) did (**Figure 1** **, panel b and** **Table 2**).

To determine whether a clone with two gene fragments in orientation would result in complementation of *aarA*, the two genes encoding MAP2426c and MAP2425c were PCR-amplified with the primer pair used in amplification of the MAV_1554-encoding gene (approx. 950 bp). PCR products were sequence-confirmed and cloned ‘in-frame’ into pBCSK. The recombinant plasmid was transformed into *P. stuartii* (XD37.A), but again, *aarA* complementation was not observed (**Figure 1** **, panel b and** **Table 2**).

Following sequence analysis, it was found that the most probable reason for the failed complementation by the MAP rhomboid-encoding genes could be absence of the W90 codon. A nonsense mutation at the W90 codon splitting the progenitor for the MAP rhomboids into two hypothetical proteins (MAP2425c and MAP2426c) was previously described [14]. A point mutation occurred at the wobble position (TGG→TGA -underlined, disrupting the tryptophan codon for a stop codon) nine base pairs upstream of the M94 codon (ATG which begins MAP2425c). While this circumvented a rhomboid pseudogene, the physiological consequence of the bifurcated proteins remains unclear (i.e. in comparison with their nearest ortholog MAV_1554 in *M. avium*, what could be the role of each of the two proteins in MAP?).

Originally annotated in strain K10 (J. Craig Venter Institute), genes encoding MAP2426c and MAP2425c were also detected in MAP isolates from Ugandan cattle with paratuberculosis (accession numbers ADO17917.1 and ADO17918.1) [14], [19].

### Use of *M. smegmatis* to decipher roles of rhomboids in mycobacteria

The saprophytic *M. smegmatis* is easy to culture and manipulate genetically and also possesses two rhomboids orthologous to those of the pathogenic MTBC species [14]. Characterizing phenotypes of rhomboid mutants of this organism provided additional insight into the roles of rhomboids in mycobacteria.

The unmarked (i.e. cells in which the antibiotic resistance genes have been removed) single and double rhomboid mutants were successfully generated through a consecutive gene deletion strategy described by Stefan et al [20]. To avoid polar mutations, in-frame deletions were designed such that approx. 100 bp of rhomboid-encoding DNA flanked upstream and downstream of the *FRT* scar that remained after unmarking.

In this text, the mutant strain in which the gene encoding MSMEG_5036 (“rhomboid protease 1”) was deleted is referred to as the Δ5036 single mutant while that in which the gene encoding MSMEG_4904 (“rhomboid protease 2”) was deleted is referred to as the Δ4904 single mutant. Likewise, the strain in which both genes encoding MSMEG_5036 and MSMEG_4904 homologs were deleted is referred to as the Δ4904Δ5036 double mutant (**Table S2**).

### Phenotypes associated with deletion of rhomboid-encoding genes in *M. smegmatis*

#### i) Effect on growth, cell and colony morphology

**Figure 2:**
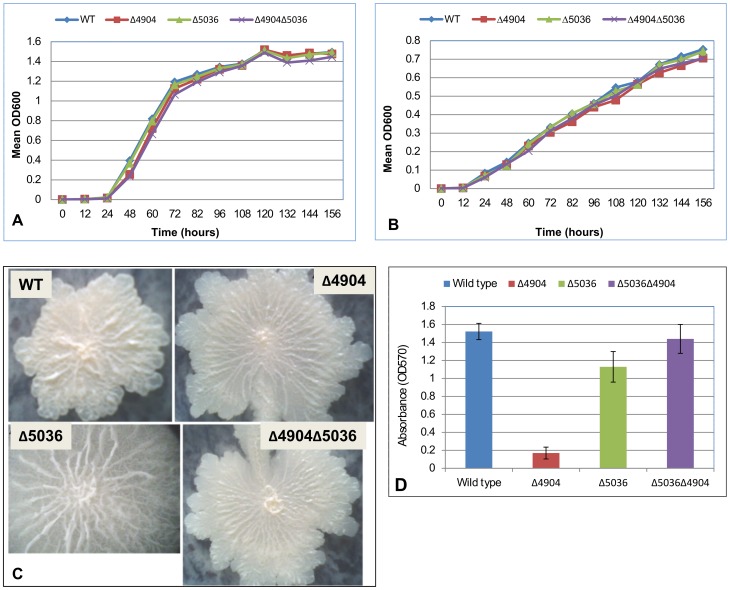
**Panels A and B:** Wild type *M. smegmatis* (WT) and rhomboid mutants (Δ4904 single, Δ5036 single and Δ4904Δ5036 double) cultured at 37°C (**Panel A**) and 42°C (**Panel B**), showing no difference in growth patterns. **Panel C:** Colony magnification showing differences in morphology between mutants (Δ4904, Δ5036 and Δ4904Δ5036) and the wild type. **Panel D:** *M. smegmatis* single rhomboid mutants were inefficient at biofilm formation. The Δ4904 single mutant (Δ4904) formed the least biofilms while the double mutant (Δ4904Δ5036) formed more biofilms than the single mutants. Each data point was from an average of four experiments.

When cultured individually for seven days, there was no difference in growth patterns between *M. smegmatis* rhomboid mutants and the wild type (p = 0.7659). This was consistent irrespective of media or temperature (**Figure 2** **, panels A and B**). Macroscopically, there was also no difference in colony morphology between mutants and the wild type. However, upon magnification of colonies, rhomboid mutants exhibited a slightly unusual morphology (**Figure 2** **, panel C**). Furthermore, for cell morphology there was no noticeable difference between mutants and the wild type.

#### ii) The single rhomboid mutants formed less biofilms

In comparison with the wild type, *M. smegmatis* single rhomboid mutants formed less biofilms (**Figure 2** **, panel D**); the Δ4904 single mutant formed the least (P<0.0001) followed by the Δ5036 single mutant (P = 0.0018). Surprisingly, the Δ4904Δ5036 double mutant formed more biofilms than the single mutants (P<0.0001). On the other hand, the difference in biofilm formation between the Δ4904Δ5036 double mutant and the wild type was not statistically significant (P = 0.3465). The possible explanation for the unusual behavior of the double mutant is discussed further below.

#### iii) The Δ4904 single mutant exhibited the lowest competitive fitness

**Figure 3:**
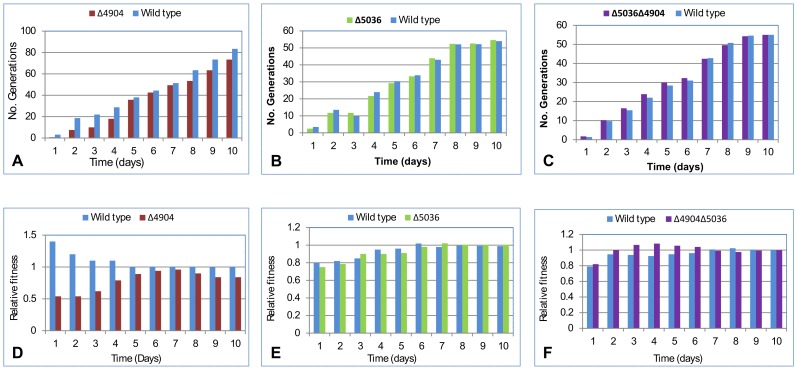
Competition and fitness assays. The Δ4904 single mutant was outcompeted by the wild type. **Panels A, B** and **C** depict the number of generations for each cell type; **Panels D, E** and **F** depict relative competitive fitness (R). The Δ4904 single mutant had the lowest R while the Δ5036 single mutant, double mutant and wild type had almost similar R values. Each data point was from an average of four experiments.

Competition assays involved cultures of rhomboid mutants mixed with the wild type and plated to determine CFUs. In these assays, the Δ4904 single mutant was outcompeted by the wild type with fewer generations and lower “relative competitive fitness” (P = 0.0003), also see **Figure 3** **, panels A and D**.

However, there was no major difference in competition assays involving the wild type and the Δ5036 single mutant (P = 0.8269); both cell types almost had similar growth patterns (**Figure 3** **, panels B and E**).

Furthermore, the Δ4904Δ5036 double mutant appeared to out-compete the wild type both in number of generations and ‘relative competitive fitness’. However, the difference was not statistically significant (P = 0.1448), also see **Figure 3** **, panels C and F**.

Overall, the Δ4904 single mutant had the lowest relative competitive fitness; 0.640 (during the first four days) and 0.851 (from day five till day 10). The relative competitive fitness was 1.198, 1.302 and 1.270 for the Δ5036 single mutant, Δ5036Δ4904 double mutant and wild type, respectively.

Taken together, the competition data suggests that the Δ4904 single mutant is physiologically less fit, implying that loss of the gene encoding MSMEG_4904 negatively affected the organism. The reverse appears true for the Δ5036 single mutant; there's no negative effect upon loss of the gene encoding MSMEG_5036. Paradoxically, the double mutant (ΔMSMEG_5036 and ΔMSMEG_4904) appears to have acquired compensatory mechanisms in that phenotypes ascribed to loss of the gene encoding MSMEG_4904 are no longer exhibited. The aberrant behavior was consistently observed irrespective of which homolog was deleted first. For mycobacteria in general, this observation may be consistent with the phenomenon of “reductive evolution and fitness” (i.e. mutations leading to genome reduction tend to favor adaptation and fitness [21], [22]). Several research groups have also reported compensatory effects following multiple gene mutations in mycobacteria [23].

#### iv) The Δ4904 single mutant was susceptible to DNA gyrase inhibitors

**Figure 4:**
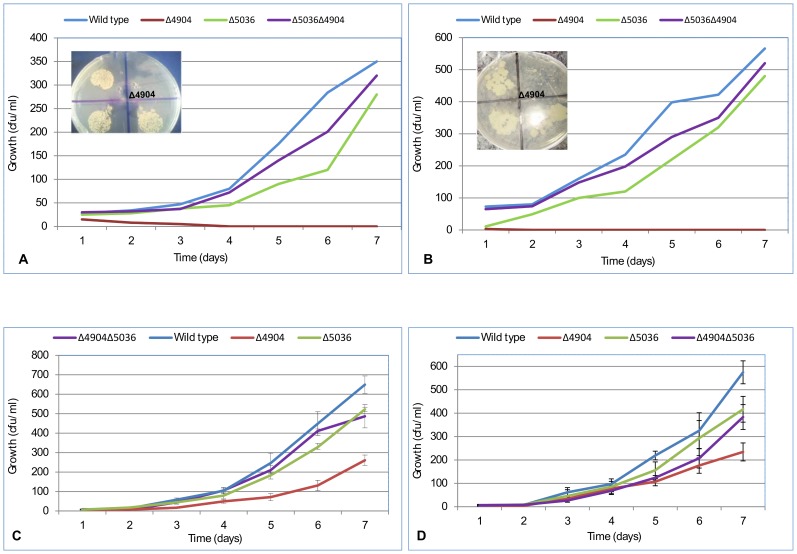
Growth inhibition assays. Depicted are rhomboid mutants (Δ4904, Δ5036 and Δ4904Δ5036) and the wild type (WT) cultured in media with 0.1 µg/ml ciprofloxacin **(panel A)**; 60 µg/ml novobiocin **(panel B)**; 100 µg/ml isoniazid **(INH, panel C)**; and 0.5 µg/ml kanamycin **(panel D)**. The Δ4904 single mutant (Δ4904) was inhibited by ciprofloxacin (0.1 µg/ml) and novobiocin (60 µg ml^−1^). In-set are similar data on solid media (7H10) showing that the Δ4904 single mutant (Δ4904) struggles to grow in presence of 0.1 µg ml^−1^ ciprofloxacin and 60 µg ml^−1^ novobiocin. Each data point was from an average of four experiments.

In comparison with the wild type, *M. smegmatis* rhomboid mutants were more susceptible to ciprofloxacin, novobiocin, isoniazid (INH) and kanamycin (**Figure 4**). Antimicrobial susceptibility was more pronounced with drugs that inhibit DNA gyrase (ciprofloxacin and novobiocin) in which the Δ4904 single mutant was completely inhibited at 0.1 µgml^−1^ and 60 µgml^−1^ (P = 0.0023 and 0.0158, respectively). In contrast, the Δ5036 single mutant was less susceptible to these drugs (P = 0.3609 and 0.3941, respectively), as well as the double Δ4904Δ5036 mutant, which was even less susceptible growing at concentrations where the Δ4904 single mutant would not (P = 0.6705 and 0.7190, respectively). Therefore from susceptibility data, the MSMEG_4904 homolog could inhibit DNA gyrase.

Again, the susceptibility data suggests occurrence of an opposing effect in the double mutant which counteracts susceptibility of the mutant to DNA gyrase inhibitors. As discussed above, this could be due to compensatory mechanisms that offset the fitness cost for loss of the two rhomboid-encoding genes. Compensatory mutations have been described in *MTB* where a series of mutations within genes associated with drug resistance offset the fitness cost associated with mutations [23]. On the other hand, one is tempted to speculate that the ameliorated effect is a consequence of both rhomboid homologs having subtle roles in a common pathway.

### Rhomboid-encoding genes from other mycobacterial species complement *MSMEG_4904* and *MSMEG_5036*

To fully ascribe the phenotypes discussed above to deletion of rhomboid-encoding genes, two types of plasmids were employed to characterize the mutants: pMV261 (an over-expression vector) and pMV361 (an integrating vector).

First, we determined the effect on phenotype presentation when *M. smegmatis* rhomboid mutants are transformed with plasmids encoding wild type rhomboids (MSMEG_4904 and MSMEG_5036).

#### i) Transforming the Δ4904 single mutant with pMV261+MSMEG_4904 reversed phenotypes

**Figure 5:**
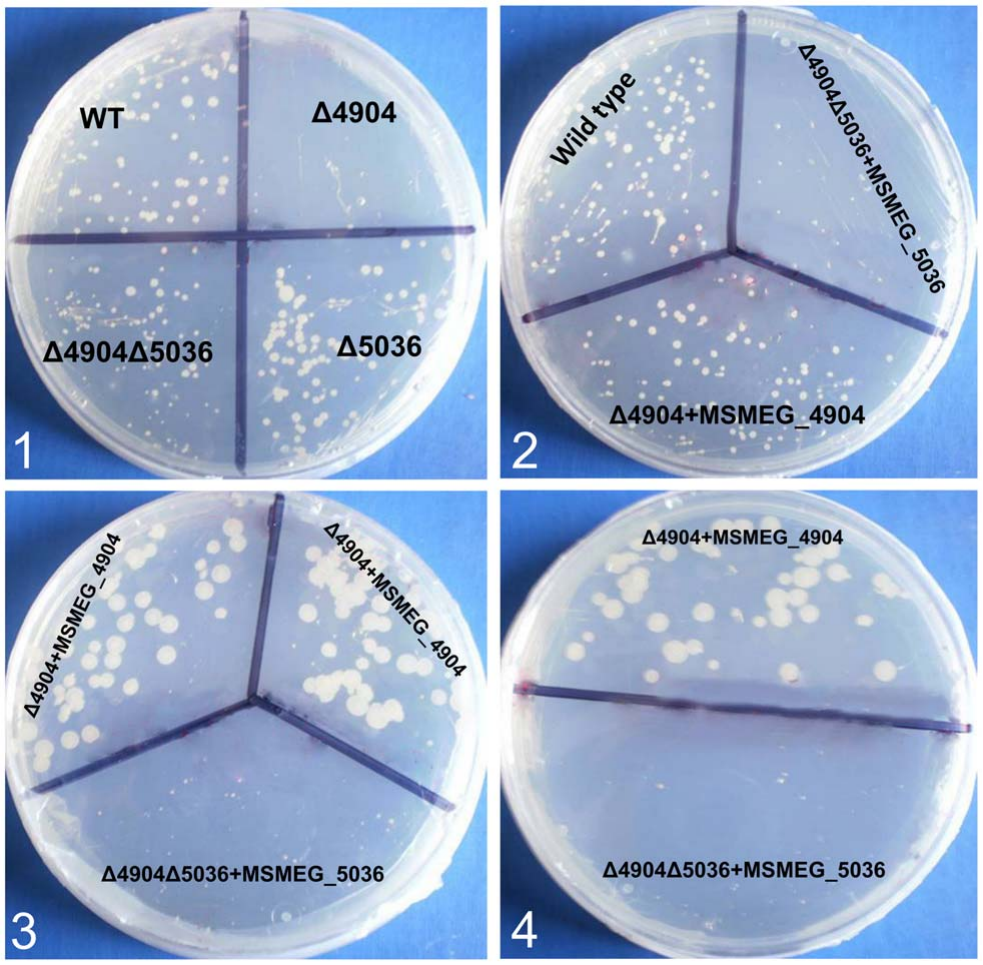
The effect of transforming *M. smegmatis* rhomboid mutants with plasmids encoding MSMEG_4904 and MSMEG_5036. **Panel 1:** shows increased susceptibility of the Δ4904 single mutant (Δ4904) to 0.1 µg ml^−1^ ciprofloxacin while the wild type (**WT**) and the Δ5036 single mutant (Δ5036) are less susceptible; the Δ4904Δ5036 double mutant (Δ4904Δ5036) was also less susceptible. **Panels 2, 3 and 4** depict the Δ4904 single mutant (Δ4904) complemented with the gene encoding MSMEG_4904 in which susceptibility to ciprofloxacin was abolished (labeled Δ4904+MSMEG_4904). When the plasmid encoding MSMEG_5036 was transformed into the double mutant (Δ5036Δ4904), a phenotype similar to that of the Δ4904 single mutant was observed in which cells were susceptible to ciprofloxacin (labeled Δ5036Δ4904+MSMEG_5036).

When the plasmid pMV261 encoding MSMEG_4904 was transformed [24] into the Δ4904 single mutant, antimicrobial susceptibility of the mutant was abolished; the phenotype of the transformed mutant was similar to that of the wild type (Figure 5, panels 2, 3 and 4). Thus, the gene encoding MSMEG_4904 fully complemented the deletion mutant. Complementation data was further verified through use of pMV361 [24] in which results were similar. This ruled out effects from over-expression of the rhomboid gene by the replicating vector (pMV261). Since phenotypes were reversed, the susceptibility of Δ4904 single mutant to DNA gyrase inhibitors can be conclusively ascribed to the deletion of the gene encoding MSMEG_4904.

In mycobacteria however, genes encoding orthologs of “rhomboid protease 2” do occur in a cluster of 10 contiguous genes, a putative operon [25]. Therefore, one may assume that deletion of the gene encoding MSMEG_4904 affected downstream genes. However, since the phenotype was reversed by transforming with a replicating vector encoding MSMEG_4904, it convincingly downplays compromising functionality of genes in this cluster. Moreover, phenotypes of the Δ4904 single mutant are ameliorated when the gene encoding a second rhomboid is deleted; if polar effects were to occur, one would expect the effect of deleting the gene encoding MSMEG_4904 to persist in the double mutant. This was not observed.

#### ii) The double rhomboid mutant became susceptible to DNA gyrase inhibitors when transformed with pMV261+MSMEG_5036

Transformation of the double mutant (Δ5036Δ4904) with pMV261+*MSMEG_5036* produced a phenotype similar to that of the Δ4904 single mutant; the transformed double mutant became susceptible to ciprofloxacin and novobiocin (**Figure 5** **, panels 2, 3 and 4**). This was puzzling. However, as discussed earlier, it could be additional evidence that deletion of a second rhomboid-encoding gene induces compensatory mechanisms.

#### iii) Rhomboid-encoding genes from MTB and MAC complemented MSMEG_4904 and MSMEG_5036

**Figure 6:**
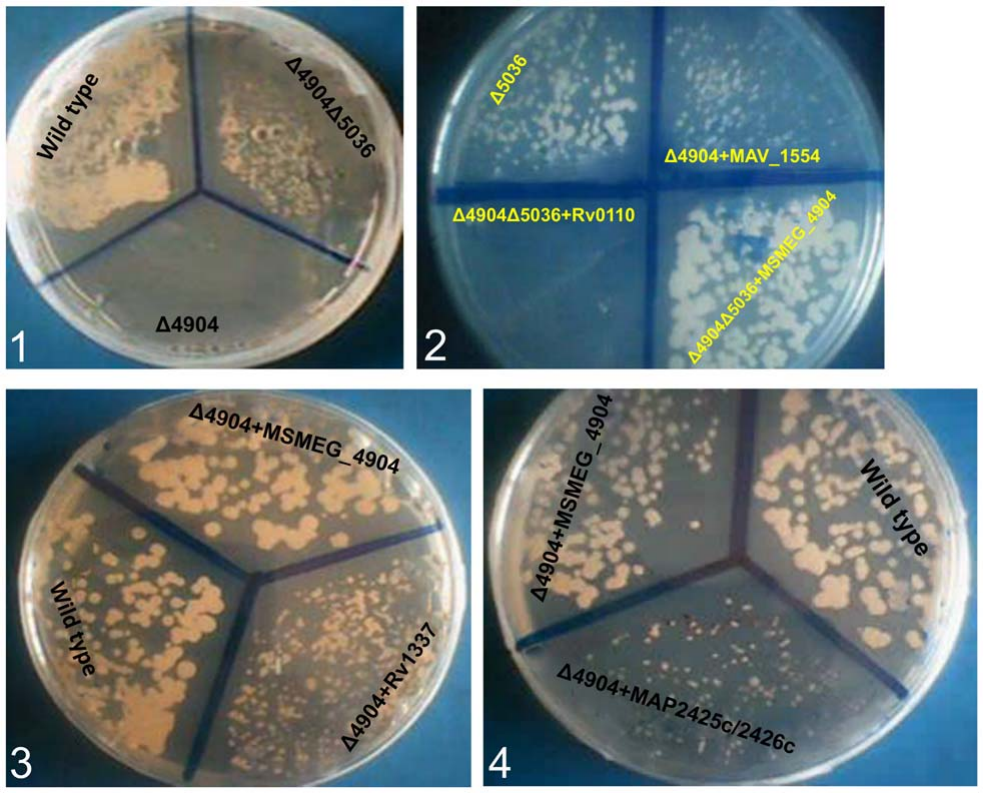
Rhomboid-encoding genes from MTB and MAC complement MSMEG_4904 and MSMEG_5036. **Panel 1:** shows the Δ4904 single mutant (Δ4904) not growing on solid medium with 0.1 µg ml^−1^ ciprofloxacin while the wild type and the double mutant (Δ4904Δ5036) grow. **Panel 2:** When transformed into the double mutant (Δ4904Δ5036), plasmids with the gene encoding Rv0110 (orthologous to MSMEG_5036) from *MTB* produced a phenotype of the Δ4904 single mutant (labeled Δ4904Δ5036+Rv0110). Similarly, transforming the single mutant with a plasmid encoding MAV_1554 (orthologus to MSMEG_4904) of *M. avium* also restored growth of the Δ4904 single mutant on 7H10 with 0.1 µg/ml ciprofloxacin (labeled Δ4904+MAV_1554). **Panel 3:** Transforming the Δ4904 single mutant with a plasmid encoding Rv1337 (orthologous to MSMEG_4904) of *MTB* restored its growth on 7H10 with 0.1 µg/ml ciprofloxacin (labeled Δ4904+Rv1337). Similarly, transforming the Δ4904 single mutant with a plasmid encoding MSMEG_4904 restores its growth on 7H10 with 0.1 µg/ml ciprofloxacin (labeled Δ4904+MSMEG_4904). **Panel 4:** Transforming the Δ4904 single mutant with a plasmid containing both genes encoding MAP2426c and MAP2425c (orthologous to MSMEG_4904) restored its growth (albeit weakly) (labeled Δ4904+MAP2425c/2426c).

To determine whether rhomboid-encoding genes from evolutionary distant species rescue phenotypes in *M. smegmatis* rhomboid mutants, the latter were transformed with plasmids encoding rhomboids of *MTB*, *M. avium* and MAP. Indeed, following transformation, it was found that genes encoding Rv1337 and MAV_1554 fully abolish phenotypes of the Δ4904 single mutant (**Figure 6** **, panels 2 and panel 3**). Furthermore, transforming the Δ4904Δ5036 double mutant with pMV261+*Rv0110* (Rv0110 is orthologous to MSMEG_5036) produced phenotypes of the Δ4904 single mutant (**Figure 6** **, panel 2**). Thus, the rhomboid-encoding genes of *MTB* and *M. avium* complement those of *M. smegmatis*.

#### iv) Discrepancy of the genes encoding MAP2425c and MAP2426c

Electroporation of the Δ4904 single mutant with pMV261+*MAP2425c/MAP2426c* (fragment encoding both MAP2426c and MAP2425c cloned in-frame) partially abolished phenotypes of the Δ4904 single mutant (**Figure 6** **, panel 4**). Thus, a combination of these gene fragments complements the MSMEG_4904-encoding gene. This was surprising since both genes encoding MAP2425c and MAP2426c either individually or in combination did not complement *aarA*. It was therefore assumed that these genes are expressed as individual proteins with somewhat divergent roles from those of other “rhomboid protease 2” orthologs.

So, does expression of a sole protein from the two genes encoding MAP2426c and MAP2425c occur? This looks unlikely. Elsewhere however, read through mechanisms overcoming nonsense mutations during transcription have been described [26]. cDNA was also amplified from MAP RNA templates using primers that amplified a similar cDNA fragment from *M. avium* (which encodes MAV_1554) [14].

Another possibility could be moonlighting behavior. Such discrepancies (i.e. failure to restore *aarA* in *P. stuartii* yet complement phenotypes in *M. smegmatis* deletion mutant) involving genes with point mutations are exhibited by some moonlighting proteins (these perform multiple functions simultaneously) [27]. However, gene expression mechanisms between *P. staurtii* and mycobacteria are different since the organisms are distantly related. This could also explain why the MAP genes were not found to be functional in the *P. stuartii* model but partially functional in the *M. smegmatis* model.

### Conclusions

We have characterized the single and double rhomboid mutants in *M. smegmatis*, in which the data suggests that MSMEG_4904 inhibits DNA gyrase. However, despite absence of phenotypes in the Δ5036 single mutant, the ameliorated effect in the double mutant alludes to occurrence of compensatory mechanisms following deletion of both rhomboid-encoding genes. Currently the implication for this is largely unknown and may not rule out a possibility of both homologs participating in a common pathway. We have also demonstrated that rhomboid-encoding genes from mycobacteria exhibit differences in complementing *aarA*. In this regard, it is only the genes encoding “rhomboid protease 2” orthologs that fully complimented *aarA*. Additionally, a non-rhomboid domain residue -tryptophan (W90) was found essential for full complementation of *aarA* by mycobacterial “rhomboid protease 2” orthologs.

Nevertheless, there are shortcomings in this report. First, failure to complement *aarA* by genes encoding “rhomboid protease 1” does not preclude rhomboid activity. “Inactive” rhomboids lacking catalytic dyad possess enzymatic activity implying that current models may be inadequate in predicting functionality. Second, while a role for MSMEG_4094 as an inhibitor of DNA gyrase is suggested, as well as compensatory mechanisms in the double mutant, experimental validation will be required to confirm the observations.

## Materials and Methods

### Plasmids, bacterial strains and growth conditions

The DNA cloning vector used was pBCSK+ phagemid (Stratagen, La Jolla, CA, USA) propagated in *E. coli* (XL1 Blue). *P. stuartii* (strains XD37 -wild type and XD37.A -ΔaarA) were provided by Dr. Philip Rather, Emory University.

The mycobacterial suicide and unmarking vectors (pMN252 and pMN597, respectively) were provided by Dr. Michael Niederweis, University of Alabama. The mycobacterial integrating and replicating vectors (pMV361 and pMV261, respectively) were provided by Dr. Peter Sander, University of Zurich. *M. avium* subspecies *paratuberculosis* (MAP) was provided by Dr. Julius B. Okuni, College of Veterinary Medicine, Animal Resources and Biosecurity, Makerere University. *M. avium* and *M. smegmatis* (SMR5) were obtained from the Joint Clinical Research Center (JCRC), Kampala. Further description of strains and vectors is provided in **Table S2**.

Except where indicated, *E. coli*, *P. stuartii* and *M. smegmatis* were cultured in Luria Bertani (LB) broth or on LB with agar [28] while *MTB*, *M. bovis* and *M. avium* were cultured in Middlebrook 7H9 supplemented with 0.2% glycerol and 0.05% Tween 80 or on Middlebrook 7H10 supplemented with 0.2% glycerol. MAP was cultured in 7H9 and on Middlebrook 7H10 but supplemented with mycobactin J (a supplement required for optimal growth of most MAP strains).

Except where indicated, antimicrobials were used at the following concentrations [20], [29], [30], [31], [32]: hygromycin, 200 µg ml^−1^; ampicillin, 100 µg ml^−1^; carbenicillin 20 µg ml^−1^; chloramphenicol, 50 µg ml^−1^; kanamycin, 50 µg ml^−1^; novobiocin, 70 µg ml^−1^; gentamicin, 20 µg ml^−1^; isoniazid (INH), 100 µg ml^−1^; rifampicin, 6 µg ml^−1^; ethambutol, 2 µg ml^−1^; and ciprofloxacin 0.1 µg ml^−1^.

### DNA cloning

PCR primers for amplifying rhomboid-encoding genes from mycobacteria were designed based on sequences from the KEGG database (Kyoto Encyclopedia of Genes and Genomes [http://www.genome.jp/kegg/catalog/org_list.html]). The DNA sequences for genes encoding MSMEG_5036, MSMEG_4904, Rv0110, Rv1337, BCG_0143, BCG_1399, Mb0114, Mb1372, MAV_1554, MAP2425c and MAP2426c were obtained by blasting mycobacterial genomes using amino acid sequences for Rv0110 or Rv1337 as queries. The Blast P algorithm was used at default settings. Then, PCR primers were designed and analyzed with the clone manager suit 7 software (Scientific and Educational software, Cary, NC, USA). Restriction endonuclease sites were included for directional cloning into pBCSK+ (**Table S3**).

Chromosomal DNA used in PCRs was extracted from mycobacteria (*MTB*, *M. bovis* BCG, *M. bovis*, *M. avium*, MAP and *M. smegmatis*) as previously described [14]. Similarly, PCR-amplification of rhomboid-encoding genes was performed as described previously [14] with high fidelity *Taq* DNA polymerase (Roche Applied Science, Mannheim, Germany). PCR products were purified with the QIAquick PCR purification kit (Qiagin, Hilden, Germany). After restriction enzyme digestion, the PCR products were ligated with similarly digested pBCSK+ and transformed into *E. coli*. Recombinants were selected according to standard procedures [28].

Recombinant plasmids were extracted and rhomboid clones sequence-confirmed (ACGT, Wheeling, IL, USA). Plasmids were designated pBCSKrho1 (containing genes encoding orthologs of “Rhomboid protease 1”) or pBCSKrho2 (containing genes encoding orthologs of “Rhomboid protease 2”), **Table S2**.

### Complementation of rhomboid deficient *P. stuartii*

To determine whether mycobacterial rhomboid-encoding genes complement *aarA*, the *P. stuatii* null-rhomboid mutant (strain XD37.A) was transformed with pBCSKrho1 and pBCSKrho2, as previously described [15]. Transformants were selected on LB with agar, chloramphenicol and 50 µg ml^−1^ X-gal (5-bromo-4-chloro-indolyl-β-D-galactopyranoside). Then, individual colonies of transformants were sub-cultured on LB with agar, chloramphenicol and X-gal to determine whether mycobacterial rhomboid-encoding genes would restore the golden yellow pigment of colonies. XD37.A transformants were also sub-cultured on MacConkey agar to determine whether mycobacterial rhomboid-encoding genes would restore growth of *P. stuartii* XD37.A on solid media with bile salts (MacConkey agar). Correction of defects in cell morphology was determined microscopically.

#### β-galactosidase (lacZ) assays

To determine whether mycobacterial rhomboid-encoding genes complement production of an extracellular putative signal in rhomboid-deficient *P. stuartii*, conditioned medium (CM) was prepared from stationary phase cultures of XD37.A transformed with pBCSKrho1 and pBCSKrho1 as described previously [33]. *P. stuartii* wild type (strain XD37) which was used as the sensor was grown to OD_600_ of 0.25 in a variety of CMs. *lacZ* assays were performed as described previuosly [6], [33] on XD37 lysates with 2 mg ml^−1^ ONPG (ortho-Nitrophenyl-β-galactoside) as the substrate.

### Site directed mutagenesis

Site directed mutagenesis (SDM) on codons for the rhomboid family and rhomboid catalytic residues in Rv1337-encoding gene was performed with pBCSKrho2D as the template (**Figure S1, Table S2** and **Table S4**). Mutagenesis was achieved through PCR using the “Phusion SDM kit”, following the manufacture's guidelines (New England Biolabs, Ipswich, MA, USA).

Mutagenesis primers were commercially phosphorylated and PAGE (polyacrylamide gel electrophoresis) purified (IDT, Coralville, IA, USA). Details for SDM primers are provided in **Table S3**.

Mutagenesis PCR products were sequence-confirmed (ACGT, Wheeling, IL, USA) to determine successful codon mutants. Recombinant plasmids with codon mutants were re-ligated and propagated in *E. coli* through transformation. Then, individual plasmids with codon mutants were purified from *E. coli* and individually assayed in XD37.A for phenotype rescue ability (as described above).

### Deletion of rhomboid-encoding genes from *M. smegmatis*

#### i) Construction of deletion vectors

To construct the deletion vector pMN252, rhomboid-flanking DNA was PCR-amplified with primers containing unique restriction endonuclease sites [20]. The primers for the rhomboid-upstream DNA (4904UP1 & 4904UP2; 5036UP1 & 5036UP2) contained PacI (forward –UP1) and SwaI (reverse –UP2) restriction sites while those for the downstream DNA (4904DN1 & 4904DN2; 5036DN1 & 5036DN2) possessed PmeI (forward –DN1) and SpeI (reverse -DN2).

To construct the vector targeting deletion of the gene encoding MSMEG_5036 (“rhomboid protease 1”), approx. 1000 bp of upstream DNA was PCR-amplified with primers 5036UP1 and 5036UP2 while the same amount of downstream DNA was amplified with primers 5036DN1 and 5036DN2. Likewise, to construct the vector targeting deletion of the gene encoding MSMEG_4904 (“rhomboid protease 2”), approx. 1000 bp of upstream DNA was PCR-amplified with primers 4904UP1 and 4904UP2 while downstream DNA was PCR-amplified with primers 4904DN1 and 4904DN2, **Table S3**.

The PCR products were purified with Phenol/Chloroform/Isoamyl alcohol, digested with the respective restriction endonucleases and ligated with similarly digested pMN252. The resultant delivery vectors were designated pMN50 (targeting deletion of the gene encoding MSMEG_5036) and pMN49 (targeting deletion of the gene encoding MSMEG_4904).

#### ii) Transformation of *M. smegmatis*

*M. smegmatis* was transformed through electroporation as described previously [20], [34] using 5 µg each of the delivery vectors pMN49 and pMN50. Transformants were selected on 7H10 with hygromycin. To allow the secondary cross-over event to occur, five colonies were sub-cultured at 37°C for one week in plain 7H9 and plated on plain 7H10. Then, colonies were sub-cultured on 7H10 with hygromycin as well as plain 7H10 to ascertain loss of the plasmid.

Pure chromosomal DNA was extracted from transformants and homologous recombination at the right locus confirmed by PCR (using primers targeting amplification of 200 bp of the internal fragment of rhomboid-encoding DNA) and Southern blotting using DIG (Digoxigenin)-labeled rhomboid DNA as probes (Roche Applied Science, Mannheim, Germany). Subsequently, all mutants (single, double, marked or unmarked) were confirmed for loss of rhomboid-encoding DNA by sequencing (in which a unique PacI cleavage site TTAATTAA missing in mycobacteria was detected in successfully generated mutants). **Table S2** describes genotypes of the mutant strains.

#### iii) Un-marking

To unmark (removal of antimicrobial resistance genes from mutants which allows consecutive transformation of mutants with a deletion vector possessing the same marker), a single colony of the confirmed marked-single mutant (hygromycin resistant, streptomycin resistant) was electroporated with 5 µg of pML597 [35] and transformants selected on 7H10 with kanamycin. After incubating at 37°C for seven days, individual colonies were cultured in plain 7H9 broth and serial dilutions plated on plain 7H10.

To confirm loss of the hygromycin resistance marker, individual colonies were simultaneously sub-cultured on 7H10 with and without hygromycin. Colonies that did not grow on hygromycin plates were presumed unmarked single mutants (hygromycin susceptible, streptomycin resistant). These were sub-cultured in 7H9 to OD_600_ of 0.3 and re-characterized through PCR (using primers targeting amplification of 200 bp of the internal fragment of rhomboid-encoding DNA, in which unmarked cells were PCR-negative) and Southern blotting (in which there was a reduction in size at the rhomboid locus). Then, a portion of culture of the confirmed unmarked mutant was prepared for electroporation with a delivery vector targeting deletion of the second rhomboid encoding gene. The double rhomboid mutant was similarly selected and unmarked to generate a strain of marker-free double rhomboid mutant.

The unmarked strains of rhomboid mutants were designated DPK2 (Δ5036 single mutant -ΔMSMEG_5036-*FRT*, Hyg^S^); DPK4 (Δ4904 single mutant -ΔMSMEG_4904-*FRT*, Hyg^S^); and DPK6, (Δ4904Δ5036 double mutant -ΔMSMEG_4904-ΔMSMEG_5036-*FRT*, Hyg^s^).

#### iv) Complementation of *M. smegmatis* rhomboid mutants

To ascribe phenotypes to loss of rhomboid-encoding genes, the wild type rhomboid-encoding genes of *M. smegmatis* (*MSMEG_5036* and *MSMEG_4904*) were cloned into integrative vectors and electroporated into mutant strains which were studied for loss of phenotypes. To rule out effects from over-expression of rhomboid encoding genes, we initially used the integrating vector pMV361 before the pMV261 replicating vector. 5 µg ml^−1^ of the constructed vectors was electroporated into the unmarked rhomboid mutants and transformants selected as previously described [34].

### Phenotype screening

Prior to detailed investigation, *M. smegmatis* rhomboid mutants were initially evaluated in a preliminary fashion as follows: macroscopic/microscopic appearance of colonies and cells; growth patterns at various temperatures (room temperature, 37°C and 42°C); growth patterns in enriched vs. minimal media (M9); growth patterns under oxygen-rich vs. oxygen-deprived conditions; biofilm forming ability; competition assays, and antimicrobial susceptibility. Detailed analyses ensued where mutants exhibited differences (see details below). Statistical analysis was determined with the Student's t-test and a P value of <0.05 was considered statistically significant.

#### i) Biofilm assays

The biofilm forming ability of wild type and rhomboid mutants was determined with the microtitter plate method [36], which is based on the ability of bacteria to adhere to plastic wells. Briefly, 0.1 ml of PCR-grade water was inoculated into wells of a 96-well micro-titer plate (PVC, Becton & Dickinson, Franklin Lakes, NJ, USA) containing 1×10^5^ cells (wild type, Δ4904 single, Δ5036 single and Δ4904Δ5036 double mutants) suspended in 0.1 ml 0.25% Tween 80. Plates were incubated at room temperature for 14 days, after which 25 µl of 1% crystal violet was added to each well (crystal violet stains the cells but not the PVC). Then, the plates were incubated at room temperature for 15 min, rinsed vigorously four times with sterile distilled water, blotted on paper towels and scored for biofilm formation. The crystal violet adsorbed onto the wells was dissolved by adding 95% ethanol and the biofilms formed determined by measuring with a Genesys spectrophotometer (Grand Blank, MI, USA) at OD_570_.

#### ii) Competition assays

Competition assays involved mixed cultures of the following; a) wild type *M. smegmatis* (Sm^R^) with marked (i.e. strain with antibiotic resistance marker following transformation) Δ5036 single mutant (strain DPK1, ΔMSMEG_5036-*hyg-FRT*; Hyg^R^); b) wild type *M. smegmatis* with marked Δ4904 single mutant (strain DPK3, ΔMSMEG_4904-*hyg-FRT*; Hyg^R^); and c) wild type *M. smegmatis* with marked Δ4904Δ5036 double mutant (strain DPK5, ΔMSMEG_4904–ΔMSMEG_5036-*hyg*-*FRT*; *Hyg^R^*).

The starting inoculum was from mid-log phase cultures standardized to a turbidity of McFarland 3. This was inoculated into 5 ml LB broth, 7H9 or M9 broth in 15 ml centrifuge tubes. Cultures were incubated at 37°C for 10 days. At 24 hr intervals, 20 µl of the competing cultures was diluted 10–1000 fold and plated on plain LB with agar, LB with agar and streptomycin or LB with agar and hygromycin and incubated at 37°C for three to five days (7H10 was used for incubation exceeding 48 h). Then, colonies on each plate were counted and the colony forming unit (CFU) for each strain determined. The CFU for the wild type was estimated from the difference in colony counts between growths on plates with streptomycin and that on plates with hygromycin.

#### iii) Estimating “relative competitive fitness”

Fitness assays for each mutant were performed as described previously [37]. Briefly, following estimation of CFU, the number of generations (G) for each cell type was calculated using the formula: G = (logB−logA)/log2, where A is CFU/ml at time zero and B is CFU/ml at a particular time point [37].

The “relative competitive fitness” (R) of the mutants was calculated from the ratio of the number of generations of mutants to that of the wild type [37]. Similarly, “R” for wild type was calculated from the ratio of the number of generations of wild type to that of mutants.

#### iv) Drug susceptibility tests

Drug susceptibility tests (DSTs) were performed by inoculating 50 µl of mid-log phase cultures (standardized to a turbidity of McFarland 3) into 3 ml of LB or 7H9. DSTs were simultaneously performed on solid media (LB with agar or 7H10) with serially diluted antimicrobial concentrations. Plates were incubated at 37°C and CFU for each cell type determined as described above.

## Supporting Information

Figure S1 **Rhomboid family residues in Rv1337 codons of which were inactivated through site directed mutagenesis.** In bold face: three histidine residues (H87, H92 and H204) conserved at the C-termini of most rhomboids; the rhomboid catalytic dyad (S149 and H204, in blue); the putative active site stabilizing residue in mycobacteria (F153); and the other rhomboid domain residues (N96, G104, G147, A148, G150, G154, G203, and G207). Also depicted are the residues (W90 and G211) appearing unique to mycobacterial rhomboids. In red or blue are residues found essential for full complementation of AarA activity. In green is G211 that instead promoted complementation. For SDM, alanine was substituted with arginine (A148R), phenylalanine with serine (F153S), glycine with glutamate (G104E, G147E, G150E, G154E, G203E, G207E and G211E), histidine with alanine (H87A, H92A and H204A), leucine with asparagine (L85N; L86N), asparagine with alanine (N96A) and serine with alanine (S149A). Gene translation was performed with the ExPaSy-translate server (http://web.expasy.org/translate/).
(TIFF)

Figure S2 **Rhomboid family residues in MAV_1554 of** ***M. avium*** **codons of which were inactivated through SDM; the rest as in Figure S1.**
(TIFF)

Figure S3 **Generation of** ***M. smegmatis*** **single and double rhomboid gene mutants.** **Panel A:** Group (a), PCR-amplification of rhomboid-encoding DNA (*MSMEG_4904* and *MSMEG_5036*) from wild type *M. smegmatis*. (b), generation of marked single and double rhomboid mutants; the ∼2 kb hygromycin resistance gene (*HygR*) that replaced rhomboid-encoding DNA was amplified at the rhomboid loci, confirming allelic exchange at the right locus and loss of rhomboid-encoding genes from *M. smegmatis*. (c), successful unmarking (i.e. removal of *HygR*) from *M. smegmatis* rhomboid mutants. M and L are 1 Kb and 0.1 kb DNA ladders, respectively. Approx. 0.6 kb of rhomboid DNA was deleted, leaving ∼0.1 kb DNA flanking the *FRT* scar (in total ∼0.2 kb rhomboid DNA was left). **Panel B:** confirmation of loss of the rhomboid-encoding gene by southern blotting. Lanes: a, detection of rhomboid DNA in the wild type; b, increase in size at the rhomboid locus due to integration of the *HygR* gene; b′, pseudo-mutant; c, unmarked mutants showing loss of rhomboid-encoding genes and reduction in size at the rhomboid locus. Genomic DNA was digested with *BSAH1* prior to electrophoresis and vacuum transfer to nylon. **Panel C:** Complementation of *M. smegmatis* rhomboid mutants in which PCR confirmed integration of rhomboid-encoding genes (*MSMEG_4904* and *MSMEG_5036*) into mutants at the right locus. Lanes: 1 and 2, PCR-amplification of the gene encoding MSMEG_4904 and MSMEG_5036 from wild type; 3, 4, 5 and 6, PCR-amplification of the rhomboid DNA scar (∼0.3 kb) from un-complemented mutants; 9, 10, 11 and 12, PCR-amplification of rhomboid-encoding genes from complemented mutants; N, negative control, M and L 1 kb and 0.1 kb DNA ladders, respectively.
(TIF)

Table S1 **Catalogue of rhomboid family proteins from completed genomes of mycobacteria.** Homologs are categorized as “rhomboid protease 1” or “rhomboid protease 2”. Sequences were retrieved from GenBank (PSI-BLAST algorithm) using ‘Rv0110’ and ‘Rv1337’ as queries or by blasting the mycobacterial genomes with the same queries. **^a^**Where applicable; **^b^**Rhomboid protease 2 in *M. ulcerans* is missing (pseudogene); ^c^ *M. avium* subsp. *paratuberculosis.*
(XLS)

Table S2 **Vectors and bacterial strains used in this study.**
(PDF)

Table S3 **Primers used in this study.**
(PDF)

Table S4 **Mutants of Rv1337-encoding gene generated through site directed mutagenesis.**
(PDF)
