# Supplementary figures and images for: Rhomboids of Mycobacteria: Characterization Using an aarA Mutant of Providencia stuartii and Gene Deletion in Mycobacterium smegmatis
Source: PLoS One. 2012 Sep 21;7(9):e45741. doi: 10.1371/journal.pone.0045741 (PMC3448690; doi:10.1371/journal.pone.0045741)

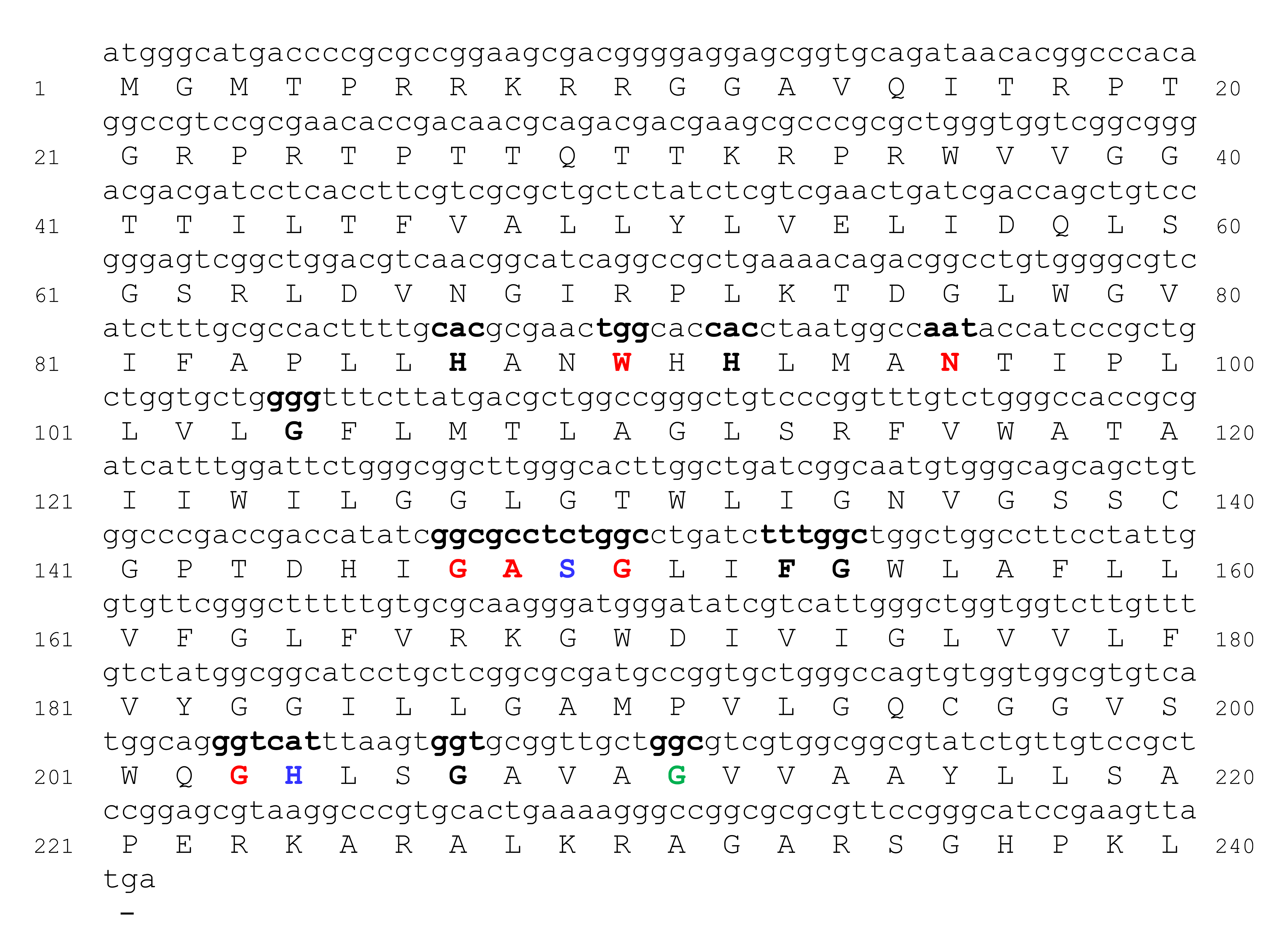

Supplement: Figure S1 — Rhomboid family residues in Rv1337 codons of which were inactivated through site directed mutagenesis. In bold face: three histidine residues (H87, H92 and H204) conserved at the C-termini of most rhomboids; the rhomboid catalytic dyad (S149 and H204, in blue); the putative active site stabilizing residue in mycobacteria (F153); and the other rhomboid domain residues (N96, G104, G147, A148, G150, G154, G203, and G207). Also depicted are the residues (W90 and G211) appearing unique to mycobacterial rhomboids. In red or blue are residues found essential for full complementation of AarA activity. In green is G211 that instead promoted complementation. For SDM, alanine was substituted with arginine (A148R), phenylalanine with serine (F153S), glycine with glutamate (G104E, G147E, G150E, G154E, G203E, G207E and G211E), histidine with alanine (H87A, H92A and H204A), leucine with asparagine (L85N; L86N), asparagine with alanine (N96A) and serine with alanine (S149A). Gene translation was performed with the ExPaSy-translate server (http://web.expasy.org/translate/). (TIFF) [file pone.0045741.s001.tif]

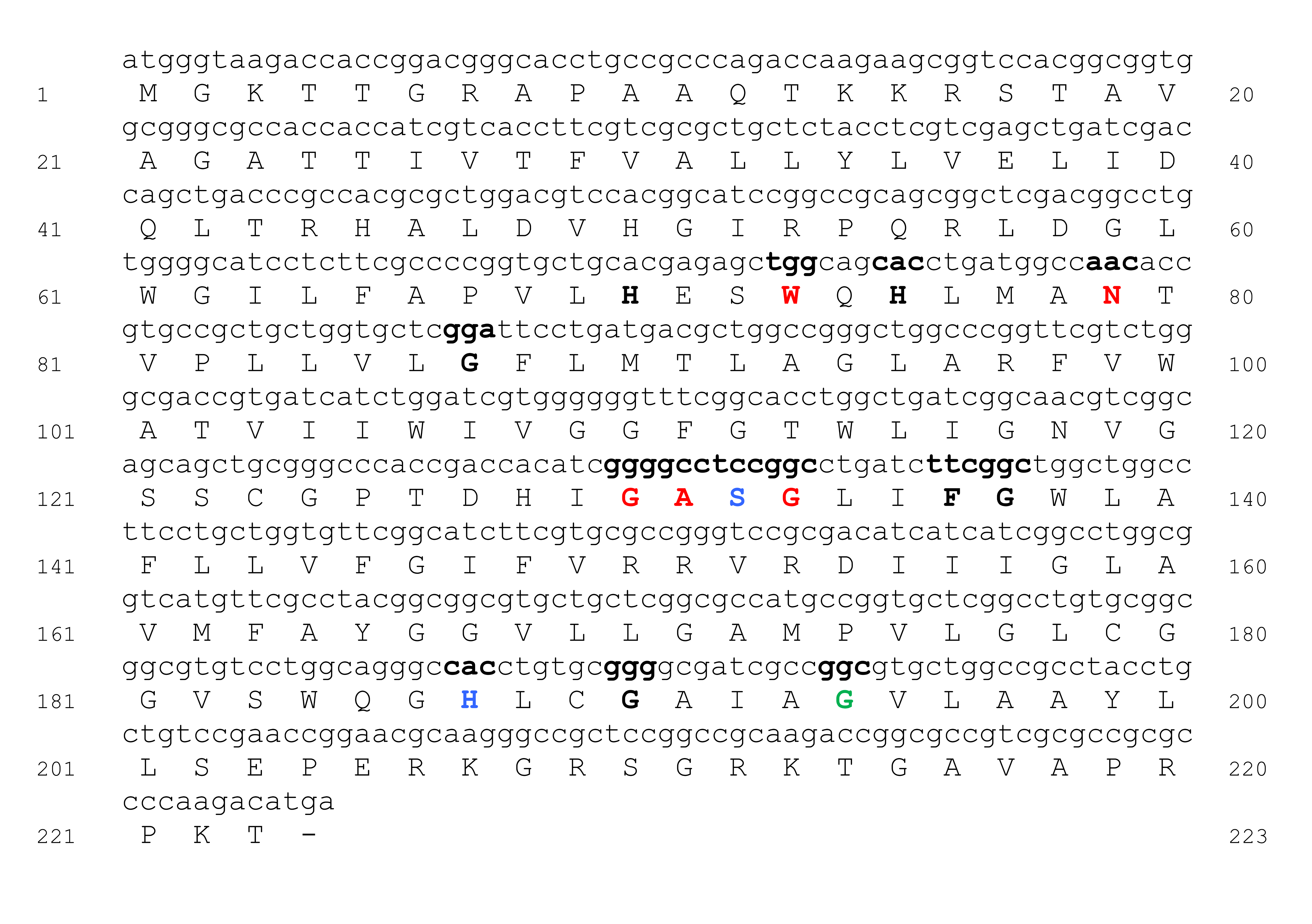

Supplement: Figure S2 — Rhomboid family residues in MAV_1554 of M. avium codons of which were inactivated through SDM; the rest as in Figure S1. (TIFF) [file pone.0045741.s002.tif]

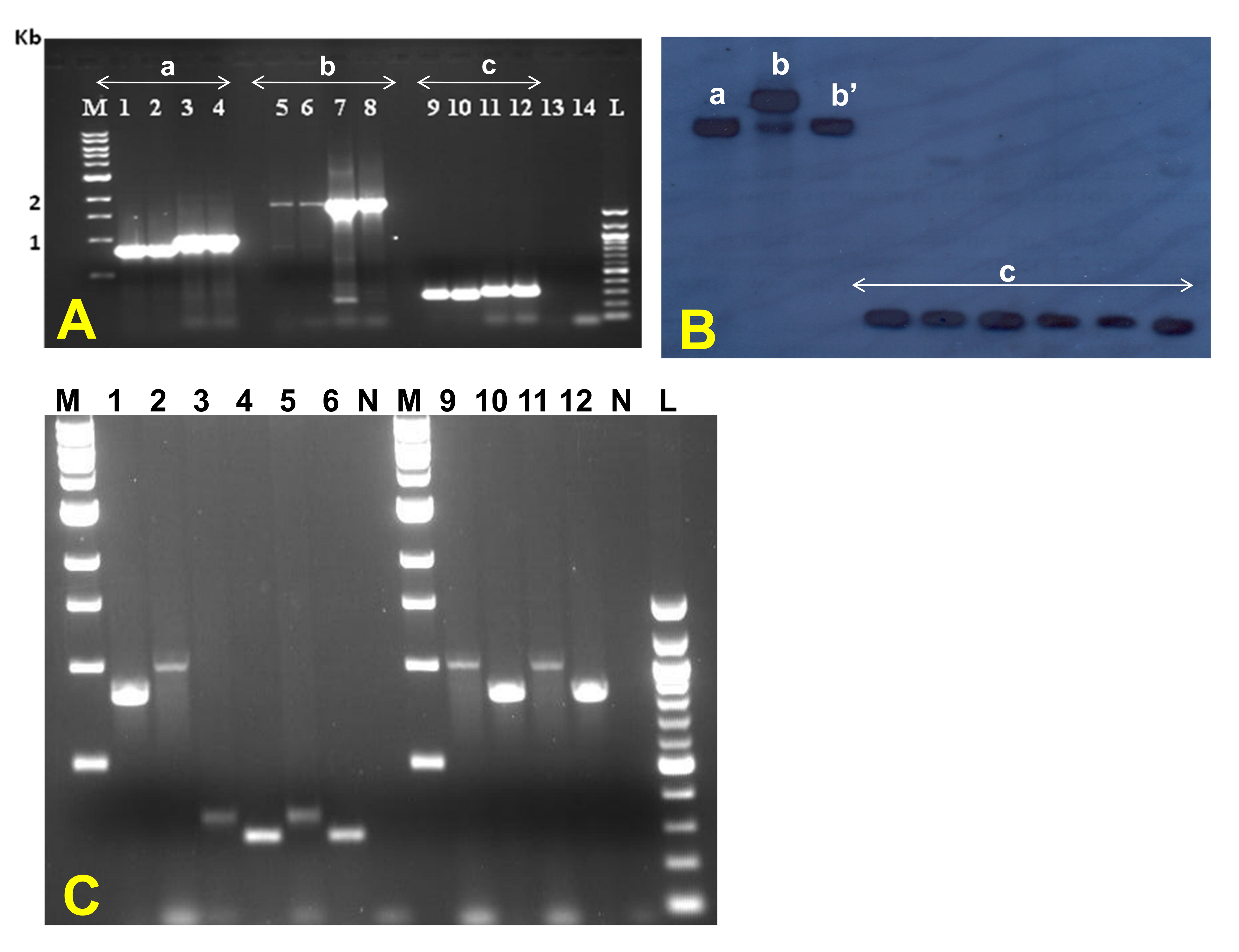

Supplement: Figure S3 — Generation of M. smegmatis single and double rhomboid gene mutants. Panel A: Group (a), PCR-amplification of rhomboid-encoding DNA (MSMEG_4904 and MSMEG_5036) from wild type M. smegmatis. (b), generation of marked single and double rhomboid mutants; the ∼2 kb hygromycin resistance gene (HygR) that replaced rhomboid-encoding DNA was amplified at the rhomboid loci, confirming allelic exchange at the right locus and loss of rhomboid-encoding genes from M. smegmatis. (c), successful unmarking (i.e. removal of HygR) from M. smegmatis rhomboid mutants. M and L are 1 Kb and 0.1 kb DNA ladders, respectively. Approx. 0.6 kb of rhomboid DNA was deleted, leaving ∼0.1 kb DNA flanking the FRT scar (in total ∼0.2 kb rhomboid DNA was left). Panel B: confirmation of loss of the rhomboid-encoding gene by southern blotting. Lanes: a, detection of rhomboid DNA in the wild type; b, increase in size at the rhomboid locus due to integration of the HygR gene; b′, pseudo-mutant; c, unmarked mutants showing loss of rhomboid-encoding genes and reduction in size at the rhomboid locus. Genomic DNA was digested with BSAH1 prior to electrophoresis and vacuum transfer to nylon. Panel C: Complementation of M. smegmatis rhomboid mutants in which PCR confirmed integration of rhomboid-encoding genes (MSMEG_4904 and MSMEG_5036) into mutants at the right locus. Lanes: 1 and 2, PCR-amplification of the gene encoding MSMEG_4904 and MSMEG_5036 from wild type; 3, 4, 5 and 6, PCR-amplification of the rhomboid DNA scar (∼0.3 kb) from un-complemented mutants; 9, 10, 11 and 12, PCR-amplification of rhomboid-encoding genes from complemented mutants; N, negative control, M and L 1 kb and 0.1 kb DNA ladders, respectively. (TIF) [file pone.0045741.s003.tif]
