# Supplementary material for: Rhomboids of Mycobacteria: Characterization Using an aarA Mutant of Providencia stuartii and Gene Deletion in Mycobacterium smegmatis
Source: PLoS One. 2012 Sep 21;7(9):e45741. doi: 10.1371/journal.pone.0045741 (PMC3448690; doi:10.1371/journal.pone.0045741)
Supplement: Table S2 — Vectors and bacterial strains used in this study. (PDF) [file pone.0045741.s005.pdf]

Table S2: Vectors and bacterial strains used in this study

**Plasmid/strain**	**Genotype/description**	**Source/reference**
**Plasmids**		
pBCSK+	Phagemid, cloning vector	Stratagene
pBCSKrho1A	pBCSK+MSMEG_5036 (Rhomboid protease 1 from *M. smegmatis* Mc2155 SMR5)	This study
pBCSKrho2B	pBCSK+MSMEG_4904 (Rhomboid protease 2 from *M. smegmatis* Mc2155 SMR5)	This study
pBCSKrho1C	pBCSK+Rv0110 (Rhomboid protease 1 from *MTB* $H_{37}Rv$ *rps*L+)	This study
pBCSKrho2D	pBCSK+Rv1337 (Rhomboid protease 2 from *MTB* H37Rv *rps*L+)	This study
pBCSKrho1E	pBCSK+Rv0110 (Rhomboid protease 1 from *MTB* strain BN44)	This study
pBCSKrho2F	pBCSK+Rv1337 (Rhomboid protease 2 from *MTB* strain BN44)	This study
pBCSKrho1G	pBCSK+BCG_0143 (Rhomboid protease 1 from *M. bovis* BCG)	This study
pBCSKrho2H	pBCSK+BCG_1399 (Rhomboid protease 2 from *M. bovis* BCG)	This study
pBCSKrho1I	pBCSK+Mb0114 (Rhomboid protease 1 from *M. bovis,* cattle strain JN55)	This study
pBCSKrho2J	pBCSK+Mb1372 (Rhomboid protease 2 from *M. bovis* cattle strain JN55)	This study
pBCSKrho2K	pBCSK+MAV1554 (Rhomboid protease 2 from *M. avium,* clinical strain SU-36800)	This study
pBCSKrho2L	pBCSK+MAP2426c (hypothetical protein without rhomboid domains; similar to N-terminus of MAV_1554)	This study
pBCSKrho2M	pBCSK+MAP2425c (hypothetical protein with rhomboid domains; similar to C-terminus of MAV_1554)	This study
pBCSKrho2N	pBCSK+MAP2425c/MAP2426c (in-frame clone with MAP2426c and MAP2425c)	This study
pMN252	Suicide vector for *rpsL*+ cells; ColE1 origin, *FRT-hyg-FRT*, *rpsL*; Amp^R; Hyg^R; 4642bp	[1]
pMN49	Suicide delivery vector for deleting MSMEG_4904; pMN252 derivative; MSMEG_4904up and MSMEG_4904down; 6416bp	This study
pMN50	Suicide delivery vector for deleting MSMEG_5036; pMN252 derivative; MSMEG_5036up and MSMEG_5036down; 6635bp	This study
pML597	Unmarking vector for *rpsL*+ cells; pMN210 derivative; flp_m; 6627bp	[2]
pMV361	Mycobacterial integrating vector	[3]
pMV261	Mycobacterial replicating vector	[3]
**Bacterial strains**		
XL1 Blue MRF'	*E. coli, recA1 endA1 gyrA96 thi-1 hsdR17 supE44*	Stratagene

	relA1 D(*lac-pro*) [F9 *proAB lacI*q *lacZ*DM15 Tn*10*]	
XD37	*P. stuartii,* PR50 *cma37*::mini-Tn*5 lacZ1*; wild type for *aarA*	[4]
XD37.A	XD37 derivative*,* Δ*aarA*	[4]
SMR5	*M. smegmatis*, mc^{2}155 derivative; Sm^R	JCRC
DPK1	SMR5 derivative; ΔMSMEG_5036-*hyg-FRT*; Hyg^R; single rhomboid mutant (marked)	This study
DPK2	SMR5 derivative; ΔMSMEG_5036-*FRT,* Hyg^S; single rhomboid mutant (unmarked)	This study
DPK3	SMR5 derivative; ΔMSMEG_4904-*hyg-FRT*; Hyg^R, single rhomboid mutant (marked)	This study
DPK4	SMR5 derivative; ΔMSMEG_4904-*FRT,* Hyg^s, single rhomboid mutant (unmarked)	This study
DPK5	SMR5 derivative; ΔMSMEG_4904-ΔMSMEG_5036-*hyg-FRT* ; *Hyg^R*, double rhomboid mutant (marked)	This study
DPK6	SMR5 derivative; ΔMSMEG_4904-ΔMSMEG_5036-*FRT,* Hyg^s , double rhomboid mutant (unmarked)	This study
H37Rv+rpsL	*MTB* $H_{37}Rv$, Sm^R derivative	[5]
BN44	*MTB* Uganda genotype strain	[6]
BCG+rpsL	*M. bovis* BCG, Sm^R	[5]
JN55	*M. bovis* cattle isolate	[7]
M. avium	*M. avium*, strain SU-36800	JCRC
MAP7	MAP cattle isolate	[8]

MAP, *Mycobacterium avium* subsp. *paratuberculosis;* SM^R, streptomycin resistant; Hyg^R, hygromycin resistant; Hyg^s, Hygromycin susceptible; Amp^R, ampicillin resistant

Additional references

1. Stephan J, Stemmer V, Niederweis M (2004) Consecutive gene deletions in Mycobacterium smegmatis using the yeast FLP recombinase. Gene 343: 181-190.
2. Song H, Niederweis M (2007) Functional expression of the Flp recombinase in Mycobacterium bovis BCG. Gene 399: 112-119.
3. Stover CK, de la Cruz VF, Fuerst TR, Burlein JE, Benson LA, et al. (1991) New use of BCG for recombinant vaccines. Nature 351: 456-460.
4. Rather PN, Ding X, Baca-DeLancey RR, Siddiqui S (1999) Providencia stuartii genes activated by cell-to-cell signaling and identification of a gene required for production or activity of an extracellular factor. J Bacteriol 181: 7185-7191.
5. Sander P, Springer B, Böttger EC (2001) Gene Replacement in Mycobacterium tuberculosis and Mycobacterium bovis BCG Using rpsL as a Dominant Negative Selectable Marker. In: Parish T, Stoker NG, editors. Mycobacterium tuberculosis Protocols: Humana Press. pp. 93-104.
6. Asiimwe BB, Koivula T, Kallenius G, Huard RC, Ghebremichael S, Asiimwe J, Joloba ML (2008) Mycobacterium tuberculosis Uganda genotype is the predominant cause of TB in Kampala, Uganda. The International Journal of Tuberculosis and Lung Disease 12: 386-391.
7. Asiimwe BB, Asiimwe J, Kallenius G, Ashaba FK, Ghebremichael S, et al. (2009) Molecular characterisation of Mycobacterium bovis isolates from cattle carcases at a city slaughterhouse in Uganda. Veterinary Record 164: 655-658.

8. Okuni JB, Dovas C, Loukopoulos P, Bouzalas I, Kateete DP, et al. (2012) Isolation of Mycobacterium avium subspecies paratuberculosis from Ugandan cattle and strain differentiation using optimised DNA typing techniques. BMC Veterinary Research 8: 99.
