# Supplementary material for: Rhomboids of Mycobacteria: Characterization Using an aarA Mutant of Providencia stuartii and Gene Deletion in Mycobacterium smegmatis
Source: PLoS One. 2012 Sep 21;7(9):e45741. doi: 10.1371/journal.pone.0045741 (PMC3448690; doi:10.1371/journal.pone.0045741)
Supplement: Table S3 — Primers used in this study. (PDF) [file pone.0045741.s006.pdf]

Table S3: Primers used in this study

**Name**	**Sequence (5'→ 3')**	**Gene ID/ description**	**Size (bp)**
**PCR-primers used for directional cloning rhomboids into pBCSK+**			
0110F	GATTACTGCAGATATTCGGCTTCGCCGGAACC (PstI)	Rv0110	869
0110R	GAGGATCTAGAACGCGAAGACAAGCGGCTATC (XbaI)	BCG_0143	869
		Mb0114	869
1337F	GATTACCCGGGACGCCGGGTGGAAGTATCTG (SmaI)	Rv1337	869
1337R	GATTAGCGGCCGCCCGACGCCGGAATCAAAGACTC (NotI)	BCG_1399	869
		Mb1372	896
1554F	GATTACCCGGGTCGACGGTGACACCGTGTTC (SmaI)	MAV_1554	954
1554R	GATTAGCGGCCGCTGCCGAGCTCATGTCTTGGG (NotI)	MAP2c[a]	950
2425cF	CGGAATTCCCACGGCATCCGGCCGCAGCGGGTCG (EcoRI)	MAP2425c	450
2425cR	GACTGCAGGGCGCCAGTGCCGAGCTCATGTC (PstI)		
2426cF	CGGAATTCCCGCCGGCATCCCGAGCTACCGG (EcoRI)	MAP2426c	269
2426cR	GACTGCAGTGGCCATCAGGTGCTGTCAGCTCTC (PstI)		
4904F	GATTACCCGGGACGCCGGATGGAAGTATCTG (SmaI)	MSMEG_4904	845
4904R	GATTAGCGGCCGCACACCGGAATCGAAGATCCC (NotI)		
5036F	GCGCAGAATTCACGGCCGGGTGAGACAAATC (EcoRI)	MSMEG_5036	1000
5036R	GCGGTACTAGTTGGACCCGGACAACATCCTG (SpeI)		
**Site directed mutagenesis primers**			
1337H87F	TTTGCGCCACTTTTGGCCGCGAACTGGCACCAC	Rv1337 (H87A)	4200
1337H87R	GATGACGCCCCACAGGCCGTCTG		
1337H92F	CACGCGAACTGGCACGCCCTAATGGCCAATACC	Rv1337 (H92A)	4200
1337H92R	CAAAAGTGGCGCAAAGATGACGCCCCACAGG		
1337W90F	CTTTTGCACGCGAACGAGCACCACCTAATGG	Rv1337 (W90E)	4200
1337W90R	TGGCGCAAAGATGACGCCCCAC		
1554W90F	GCACGAGAGCTGGCAGCACCTGATG	MAV1554 (W90E)	
1554W90R	AGCAGCGGCACGGTGTTGGC		
1337N96F	CACCACCTAATGGCCGCCACCATCCCGCTGCTG	Rv1337 (N96A)	4200
1337N96R	CCAGTTCGCGTGCAAAAGTGGCGCAAAG		
1337G104F	CCGACCGACCATATCGAGGCCTCTGGCCTGATC	Rv1337 (G104E)	4200
1337G104R	GCCACAGCTGCTGCCCACATTGCCGATCAGCCAAG		
1337G147F	CATATCGGCGCCTCTGAGCTGATCTTTGGCTGGC	Rv1337 (G147E)	4200
1337G147R	GTCGGTCGGGCCACAGCTGCTG		
1337A148F	CGACCGACCATATCGGCCGATCTGGCCTGATCTTTGGC	Rv1337 (A148R)	4200
1337A148R	GGCCACAGCTGCTGCCCACATTGC		
1337S149F	GACCATATCGGCGCCGCCGGCCTGATCTTTGGC	Rv1337 (S149A)	4200
1337S149R	GGTCGGGCCACAGCTGCTGCCCAC		
1337G150F	TCTGGCCTGATCTTTGAGTGGCTGGCCTTCCTATTG	Rv1337 (G150E)	4200
1337G150R	GGCGCCGATATGGTCGGTCGGGCCACAG		
1337F153F	GCCTCTGGCCTGATCTCGGGCTGGCTGGCCTTC	Rv1337 (F153S)	4200
1337F153R	GCCGATATGGTCGGTCGGGCCACAGC		
1337G154F	GTGGCGTGTCATGGCAGGAGCATTTAAGTGGTGCGGTTG	Rv1337 (G154E)	4200
1337G154R	CACACTGGCCCAGCACCGGCATC		
1337G203F	TCATTTAAGTGAGGCGGTTGCTG	Rv1337 (G203E)	4200

1337G203R	CCCTGCCATGACACGCCACCACACTG		
1337H204F	GGCGTGTCATGGCAGGGTGCCTTAAGTGGTGCGGTTGC	Rv1337 (H204A)	4200
1337H204R	ACCACACTGGCCCAGCACCGGCATC		
1337G207F	TCATTTAAGTGAGGCGGTTGCTG	Rv1337 (G207E)	4200
1337G207R	AGATACGCCGCCACGACGC		
1337G211F	AGTGGTGCGGTTGCTGAGGTCGTGGCGGCGTATC	Rv1337 (G211E)	4200
1337G211R	CTTTTTGCACGCGAACGAGCACCACCTAATGG		
**PCR-primers used in constructing suicide delivery vector pMN252**			
4904UP1	AGCTTTGTTTAAACGCTGCGGATGATCGAACAGG (PmeI)	MSMEG_4904 upstream	852
4904UP2	GGACTAGTCCGGGTCATGCCGGTTCCACGACCAC (SpeI)		
4904DN1	CCTTAATTAAGGCTGACCTCATGAGCGATCGAC (PacI)	MSMEG_4904 downstream	922
4904DN2	CCATTTAAATGGCAGGATGGTCAAACGCACAC (SwaI)		
5036UP1	AGCTTTGTTTAAACGTGAGCACACGCCGCTGATC (PmeI)	MSMEG_5036 upstream	1009
5036UP2	GGACTAGTCCCGGCGGCCGTGCGCATGCACTCCG (SpeI)		
5036DN1	CCTTAATTAAGGTAGATCGCCGCGCCCGGATTCAGG (PacI)	MSMEG_5036 downstream	984
5036DN2	CCATTTAAATGGCCCGCCGGATCCGTCGTCGTTCG (SwaI)		
4904int1	GTTGCACGCCAACTGGGAAC	MSMEG_4904 internal fragment	279
4904int2	ACACCAGCAGCACGAGGATG		
5036int1	CGTCGATGTTCCTGCACTAC	MSMEG_5036 internal fragment	372
5036int2	TGATCGCGGTTCCCAAAG		
**PCR-primers used in constructing integrating and replicating vectors (pMV361 and pMV261)**			
pMV4904F	GATTACTAGTACGCCGGATGGAAGTATCTG (SpeI)	MSMEG_4904	845
pMV4904R	GATTACGCGTACACCGGAATCGAAGATCCC (MluI)		
pMV5036F	GCGCACTAGTACGGCCGGGTGAGACAAATC (SpeI)	MSMEG_5036	1000
pMV5036R	GCGGACGCGTTGGACCCGGACAACATCCTG (MluI)		
pMV0110F	GATTACTAGTATATTCGGCTTCGCCGGAACC (SpeI)	Rv0110	967
pMV0110R	GCGGACGCGTACGCGAAGACAAGCGGCTATC (MluI)		
pMV1337F	GATTACTAGTACGCCGGGTGGAAGTATCTG (SpeI)	Rv1337	869
pMV1337R	GCGGACGCGTCCGACGCCGGAATCAAAGACTC (MluI)		
pMV1554F	GATTACTAGTTCGACGGTGACACCGTGTTC (SpeI)	MAV_1554	954
pMV1554R	GCGGACGCGTTGCCGAGCTCATGTCTTGGG (MluI)		
pMAPF	GATTACTAGTTCGACGGTGACACCGTGTTC (SpeI)	MAP2425c/MAP2426c	954
pMAPR	GCGGACGCGTTGCCGAGCTCATGTCTTGGG (MluI)		

Restriction sites to facilitate directional cloning into vectors are underlined (and described in parenthesis beside each primer). 1 or F refers to the forward primer while 2 or R refers to reverse primer. For site directed mutagenesis, codons of the inactivated/substituted residues are underlined. Unless stated otherwise, primers were synthesized by IDT, Leuven, Belgium.
