# Supplementary material for: Rhomboids of Mycobacteria: Characterization Using an aarA Mutant of Providencia stuartii and Gene Deletion in Mycobacterium smegmatis
Source: PLoS One. 2012 Sep 21;7(9):e45741. doi: 10.1371/journal.pone.0045741 (PMC3448690; doi:10.1371/journal.pone.0045741)
Supplement: Table S4 — Mutants of Rv1337-encoding gene generated through site directed mutagenesis. (PDF) [file pone.0045741.s007.pdf]

Table S4: Rv1337 mutants generated through site directed mutagenesis

Wild type position[a]	Wild type residue	Wild type codon	Mutant codon	Mutant residue	Mutant
L86	Leucine	CTG	AAT	Asparagine	Rv1337.L86N
H87	Histidine	CAC	GCC	Alanine	Rv1337.H87A
H92	Histidine	CAC	GCC	Alanine	Rv1337.H92A
W90	Tryptophan	TGG	GAG	Glutamate	Rv1337.W90E
W90[b]	Tryptophan	TGG	GAG	Glutamate	MAV1554.W90E
N96	Asparagine	AAT	GCC	Alanine	Rv1337.N96A
G104	Glycine	GGG	GAG	Glutamate	Rv1337.G104E
G147	Glycine	GGC	GAG	Glutamate	Rv1337.G147E
A148	Alanine	GCC	CGA	Arginine	Rv1337.A148R
S149	Serine	TCT	GCC	Alanine	Rv1337.S149A
G150	Glycine	GGC	GAG	Glutamate	Rv1337.G150E
F153	Phenylalanine	TTT	TCG	Serine	Rv1337.F150S
G154	Glycine	GGC	GAG	Glutamate	Rv1337.G154E
G203	Glycine	GGT	GAG	Glutamate	Rv1337.G203E
H204	Histidine	CAT	GCC	Alanine	Rv1337.H204A
G207	Glycine	GGT	GAG	Glutamate	Rv1337.G207E
G211	Glycine	GGC	GAG	Glutamate	Rv1337.G211E

[a]The position is fronted with the amino acid type (single letter code); [b]template was gene encoding MAV_1554
